# Release of hepatitis B virions is positively regulated by glucose‐regulated protein 78 through direct interaction with preS1

**DOI:** 10.1002/jmv.28271

**Published:** 2022-12-01

**Authors:** Yueyuan Shi, Xin Jin, Shuang Wu, Junye Liu, Hongpeng Zhang, Xuefei Cai, Yuan Yang, Xiang Zhang, Jie Wei, Miao Luo, Hua Zhou, Huihao Zhou, Ailong Huang, Deqiang Wang

**Affiliations:** ^1^ Key Laboratory of Molecular Biology of Infectious Diseases designated by the Chinese Ministry of Education Chongqing Medical University Yuzhong Chongqing China; ^2^ College of Laboratory Medicine Chongqing Medical University Yuzhong Chongqing China; ^3^ Department of Clinical Laboratory The People's Hospital of Yubei District of Chongqing City Yubei Chongqing China; ^4^ Department of Clinical Laboratory The Second Hospital of Harbin Harbin City Heilongjiang Province China; ^5^ Department of Clinical Laboratory The Affiliated Children Hospital of Xi'an Jiaotong University Xi'an City Shanxi Province China; ^6^ Department of Clinical Laboratory, Honghui Hospital Xi'an Jiaotong University Xi'an City Shanxi Province China; ^7^ Department of Blood Transfusion Women and Children's Hospital of Chongqing Medical University Yubei Chongqing China; ^8^ Department of Clinical Laboratory The Second Affiliated Hospital of Chongqing Medical University Yuzhong Chongqing China; ^9^ Research Center for Drug Discovery, School of Pharmaceutical Sciences Sun Yat‐sen University Guangzhou China

**Keywords:** antiviral, enveloped particles, GRP78, hepatitis B virus, peptide, virion release

## Abstract

In this study, we investigated the mechanism of hepatitis B virus (HBV)‐enveloped particle release. Specifically, we used preS1 as a bait protein to screen host proteins using mass spectroscopy, with the results of immunofluorescence, western blot, co‐immunoprecipitation, isothermal titration calorimetry, and pull‐down assays identifying glucose‐regulated protein (GRP)78 as a specific target for preS1 binding. We employed transcriptome sequencing, enzyme‐linked immunosorbent assays, and particle gel assays to investigate the mechanism of GRP78‐mediated positive regulation of HBV‐enveloped particle release. Additionally, we performed phage‐display, surface plasmon resonance, and molecular‐docking assays to assess peptides inhibiting enveloped‐particle release. We found that HBV upregulated GRP78 expression in liver cell lines and the serum of patients with chronic hepatitis B. Furthermore, GRP78 promoted the release of HBV‐enveloped particles in vitro and in vivo within an HBV transgenic mouse model. Moreover, we identified interactions of preS1 peptides with GRP78 via hydrogen bonding and hydrophobic interactions, which effectively inhibited its interaction with HBV‐enveloped particles and their subsequent release. These findings provide novel insights regarding HBV virion release, and demonstrated that GRP78 interacted with preS1 to positively regulate the release of HBV‐enveloped particles, suggesting GRP78 as a potential therapeutic target for inhibiting HBV infection.

## 1 INTRODUCTION

Hepatitis B virus (HBV) is a major human pathogen responsible for chronically infecting >250 million people worldwide.^1^, ^2^ Although a prophylactic vaccine is available, vaccinations are not administered effectively, and new infections continue to occur. Therapeutic options for chronic HBV infection, including six nucleo(s/t)ide analogs and pegylated interferon (IFN)‐α, can significantly reduce viral load and prevent liver disease progression. Although nucleoside analogs represent the most potent anti‐HBV drugs, they are also associated with drug resistance and require lifelong administration due to their inability to eradicate the virus from infected hepatocytes.^2^, ^3^ Therefore, novel therapies to cure HBV infection are required.^4^, ^5^

As an enveloped virus, HBV contains three viral surface proteins (the large, middle, and small HBV surface proteins [LHBs, MHBs, SHBs, respectively]). These integral membrane proteins are embedded in the membrane surrounding the nucleocapsid, which is assembled by the core protein (HBc) and harbors the viral DNA genome. A major characteristic of HBV is the release of various complete and incomplete viral particles from infected cells, including nucleocapsids with or without viral nucleic acids, subviral particles containing only envelope proteins, and intact viral particle virions containing an outer envelope that encloses an inner capsid with or without viral nucleic acids.^6^, ^7^ In particular, a complete HBV particle (enveloped particle) contains an outer envelope that encloses an inner capsid with relaxed circular DNA. Consequently, the processes of virion assembly and transport in the cytoplasm and enveloped‐particle release represent potential targets for antiviral drugs.^5^, ^8^

Host factors can interact with HBc and envelope proteins to participate in the release of HBV virion. For example, Vps4 and the molecular endosomal‐sorting complexes required for transport (ESCRT) cooperate in the release of HBV virions.^9^, ^10^ Moreover, cellular kinases can regulate virion formation and promote intracellular capsid accumulation by modifying capsid phosphorylation.^11^, ^12^, ^13^ However, these studies focused on the release of HBV core particles, whereas the precise mechanism underlying HBV virion release remains unclear.

Glucose‐regulated protein 78 (GRP78) is an important endoplasmic reticulum (ER) chaperone that functions in many cellular processes, including protein assembly, folding, and translocation across the ER membrane. GRP78 comprises two functional domains: the N‐terminal ATPase domain (nucleotide‐binding domain [NBD], residues 31–407) and a C‐terminal peptide‐binding domain (substrate‐binding domain [SBD], residues 4–654).^14^, ^15^, ^16^, ^17^ GRP78 plays regulatory roles in the process and function of certain viral envelope proteins, including those of the Middle East respiratory syndrome coronavirus (MERS‐CoV), Ebola virus (EBOV), Zika virus, Dengue virus, Japanese encephalitis virus (JEV), Sindbis virus, hepatitis C virus (HCV), vesicular stomatitis virus, and influenza A virus. Thus, GRP78 may serve as a receptor or cofactor to aid viral entry into host cells.^18^, ^19^ Patrick Reid et al.^20^ found that epigallocatechin gallate, which inhibits the ATPase activity of GRP78, can inhibit EBOV transcription and infection, and Nain et al.^21^ found that GRP78 plays an important role in JEV replication. Furthermore, GRP78 can reportedly inhibit HBV infection; however, the mechanism underlying this antiviral effect remains elusive.^22^, ^23^, ^24^

In this study, we demonstrated a firsthand role for direct interaction of GRP78 with the PreS1 domain to positively regulate HBV‐enveloped particle secretion in vitro and in vivo. Furthermore, screening using a phage‐display assay and identification of a hydrophobic peptide from PreS1 revealed that GRP78 binding inhibited the release of enveloped viral particles. These results provide enhance the understanding of HBV‐particle excretion and therapeutic targeting in antiviral therapy.

## 2 MATERIALS AND METHODS

### 2.1 Patients

Supporting Information: Table S1 shows the characteristics of the patients included in this study (103 women and 68 men; median age: 55.2 years [range: 18–65 years]). The study protocol was approved by the Research Ethics Committee of Chongqing Medical University, and written informed consent was obtained from each patient.

### 2.2 Cell culture, plasmids, and mice

The HepG2, HepG2.2.15, and HepAD38 cell lines were kindly provided by Professor Ninshao Xia (Wuhan University, Wuhan, China). HepG2.2.15 cells were cultured in Dulbecco's modified Eagle medium (DMEM) containing 500 μg/ml G418 (Gibco), whereas HepG2 and HepAD38 cells were cultured in DMEM alone. All culture media were supplemented with 10% (v/v) fetal calf serum (Thermo Fisher Scientific) at 37°C under 5% CO_2_ atmosphere.

The HBV‐replication plasmids (pcDNA3.1‐HBV1.1) and the recombinant plasmids expressing GRP78 and preS1 were preserved in the laboratory. Adenoviruses expressing green fluorescent protein (GFP), wild‐type (WT) GRP78, and their mutants were constructed and prepared.

Adenovirus mixed with 5 × 10^9^ GFU of pAd‐GRP78 or pAd‐GFP was dissolved in 0.3 ml of saline and injected into HBV transgenic mice (TgM; male, 6–8‐week‐old; provided by Prof. Ning‐shao Xia, School of Public Health, Xiamen University) via the tail vein over a 10‐s period, respectively. Serum was collected and liver tissue harvested at 4 weeks after viral injection. All animal studies were performed in accordance with protocols approved by the Rules for Animal Experiments published by the Chinese Government and the Research Ethics Committee of Chongqing Medical University (February 26, 2021).

### 2.3 Preparation of virus particles by sucrose density gradient ultracentrifugation

Sucrose density gradient ultracentrifugation was performed as previously described, with slight modifications. Briefly, discontinuous sucrose density gradients (10%, 20%, 30%, 40%, 50%, and 60%) were prepared with a phosphate‐buffered saline (PBS) solution. HepAD38 culture supernatant was laid on the sucrose gradients and centrifuged at 1.12 × 10^5^ *g* for 15 h at 10°C using a SW28 rotor (Beckman Coulter). The precipitate with 30% sucrose density gradients was slowly dissolved with PBS overnight and used for particle gel, western blot, Southern blot, and quantitative polymerase chain reaction (qPCR) experiments for viral particle detection.

### 2.4 Particle gel assay

HBV virions were identified by particle gel assay.^25^ HBV‐producing cells were cultured, followed by harvesting of the culture medium. Cell supernatant was added to 35% PEG8000 and shaken at 4°C for >2 h. The viral particles were dissolved in TNE buffer, incubated overnight at 4°C, and subjected to agarose gel electrophoresis. The samples were transferred to a nitrocellulose membranes, treated with methanol for 30 min, blocked with 5% nonfat milk, and incubated with anti‐preS1 (for enveloped particles) or anti‐HBc (for capsid particles) antibodies for 1 h at 20°C. Following incubation with an antimouse secondary antibody (1:10,000) at 4°C overnight, the membrane was visualized using the an enhanced chemiluminescence western blot detection kit (Beyotime Biotech).

### 2.5 Molecular docking

To calculate the binding energies between GRP78 and the peptide molecule, we performed molecular docking to screen the orientation pattern of the peptides in the binding groove of the GRP78 molecules and identify the residues involved in the interaction. The molecular structure of GRP78 was downloaded from the RCSB Protein Data Bank (PDB: 6HAB; http://www.rcsb.org/structure/6HAB), and docking analysis was performed using AutoDock (v4.2; http://autodock.scripps.edu/). The binding model with the best energy score was selected for subsequent analysis. The images used to visualize interactions between GRP78 and the peptide were generated using Pymol (https://pymol.org/2/).

### 2.6 Statistical analysis

Data are presented as the mean ± standard error of the mean (SEM) of at least three independent experiments. All statistical analyses were performed using SPSS (v19.0; IBM Corp.) and GraphPad Prism software (v8.4; GraphPad Software). Student's *t‐*test was used to compare results between two groups, with a *p* < 0.05 considered statistically significant.

## 3 RESULTS

### 3.1 Screening of host proteins that interact with preS1

To identify host proteins that interact with PreS1,^28^ we prepared a recombinant glutathione S‐transferase (GST)‐preS1 protein as bait and detected specific proteins with a molecular weight between 97 and 116 kDa in HepG2 cell lysate, including keratin type II skeleton 1, type I skeleton 9, LanC‐like protein 1 (LanCL1), and GRP78, using mass spectrometry (Supporting Information: Figure S1). LanCL1, a peptide‐modifying enzyme component in eukaryotic cells, serves as a glutathione transferase and is reportedly associated with breast cancer and prostate cancer.^26^ Furthermore, GRP78 can be galactosylated at three residues, participates in viral entry into the host cell, and/or regulates the replication of viruses, including MERS‐CoV, JEV, EBOV, HCV, and HBV.^18^, ^19^, ^27^, ^28^, ^29^

**Figure 1:**
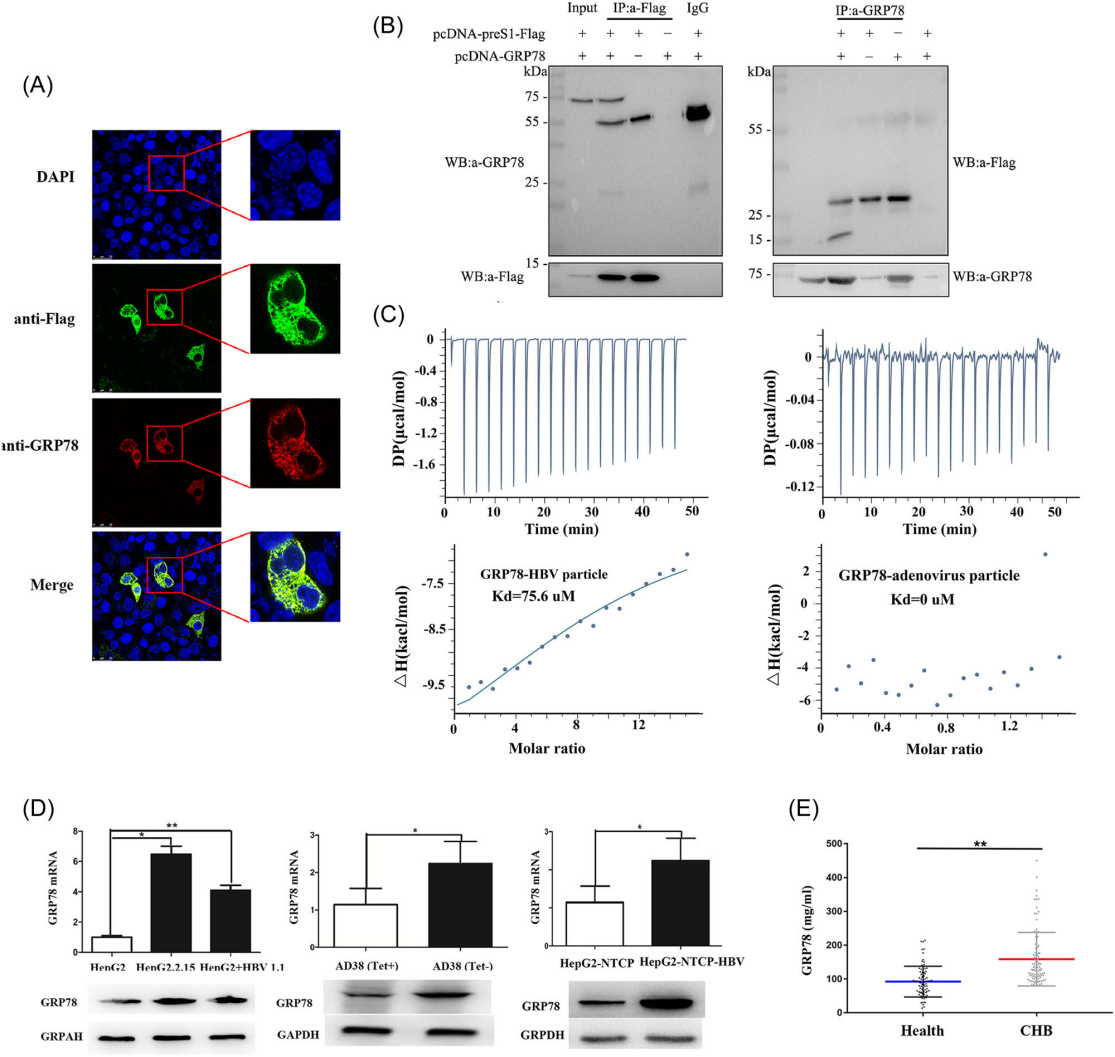
Direct interaction between HBV preS1 and GRP78. (A) Immunofluorescence confocal detection of GRP78 and preS1 in HepG2.2.15 cells. (B) Immunoprecipitation of GRP78 and Flag‐preS1. (C) GRP78 interacts with HBV particles according to ITC analysis, with adenovirus particles used as controls. (D) GRP78 expression in HepG2, HepAD38, and HepG2‐NTCP cells (*p < 0.05, **p < 0.01 [*n* = 3]). (E) Quantification of GRP78 level (**p < 0.01 [*n* = 92 or 79]). GRP78, glucose‐regulated protein 78; HBV, hepatitis B virus; ITC, isothermal titration calorimetry; NTCP, Na⁺‐taurocholate co‐transporting polypeptide.

Confocal immunofluorescence detection further revealed that both GRP78 and preS1 were spatially colocalized and primarily distributed throughout the cytoplasm (Figure 1A). Additionally, co‐immunoprecipitation revealed an interaction between GRP78 and preS1 in HepG2 cells (Figure 1B). To verify this interaction, we expressed and purified three truncated versions of GST‐preS1 (preS1‐p1 [residues 1–65], preS1‐p2 [residues 25–90], and preS1‐p3 [residues 66–119]). The results of microscale thermophoresis assays revealed that these truncated proteins interacted directly with GRP78 with high affinity (Kd = 899 ± 18.8 nM, 1200 ± 51.3 nM, and 431 ± 20.1 nM, respectively) (Supporting Information: Figure S2). These results suggested that preS1 contains multiple binding sites for GRP78. Consequently, we further evaluated the interaction between GRP78 and preS1 in the HBV life cycle.

We subsequently identified significant increases in both GRP78 mRNA and protein levels (>2‐fold) in HepG2.2.15 and HepG2 cells transfected with HBV plasmids relative to levels in HBV‐negative cells (*p* < 0.05), with similar results observed in HepAD38 cells treated with or without tetracycline (Figure 1D). Additionally, infecting HepG2‐ Na⁺‐taurocholate co‐transporting polypeptide (NTCP) cells with HBV increased GRP78 expression (Figure 1D and Supporting Information: Figure S3). To further investigate the relationship between GRP78 and HBV, we detected GRP78 expression in 171 clinical serum samples from patients with chronic hepatitis B infection (Supporting Information: Table S1). We found that the average serum GRP78 concentration (125.1 mg/ml, *n* = 92) of chronic HBV carriers was higher than that in healthy individuals (92.31 mg/ml, *n* = 79; *p* < 0.01) (Figure 1E). Moreover, detection of molecular markers of the unfolded protein response revealed that in addition to GRP78 and protein kinase R‐like ER kinase (PERK), the expression levels of eukaryotic translation‐initiation factor 2α, activating transcription factor 4, inositol‐requiring enzyme 1α (IRE1α) and X‐box‐binding protein 1 were consistently elevated following HBV overexpression in HepG2 cells (Supporting Information: Figure S3). Therefore, HBV expression may activate the PERK and IRE1α pathways to induce the ER stress response.^30^, ^31^

Additionally, we collected purified recombinant GRP78 protein (Supporting Information: Figure S2) and HBV‐enveloped particles from the cell supernatant, followed by separation by ultracentrifugation at 4°C for isothermal titration calorimetry (ITC) experiments. We found that GRP78 interacted with HBV‐enveloped particles (Kd = 75.6 × 10^−6^ M) (Figure 1C), whereas minimal interactions were observed between GRP78 and adenovirus. These findings confirmed the ability of HBV to promote GRP78 expression and identified that GRP78 directly interacts with preS1 and HBV‐enveloped particles.

### 3.2 GRP78 promotes HBV virion release

preS1 is the viral‐binding site for NTCP, the HBV receptor, and a unique antigen presented on the outermost surface of the HBV‐enveloped particle.^22^ However, GRP78 binding to preS1 did not significantly affect viral entry into host liver cells (data not shown). Transcriptome sequencing data for pAd‐GRP78infected HepG2.2.15 cells (Supporting Information: Figure S4) revealed activation of that multiple pathways associated with gene transcription (DNA replication, RNA degradation, and RNA transport) and cancer (p53 signaling and the cell cycle), with the most enriched pathway including proteins associated with protein export and trans‐shipment (Supporting Information: Figure S5). These findings provided insights suggesting that GRP78 might affect the release of HBV‐enveloped particles.

**Figure 2:**
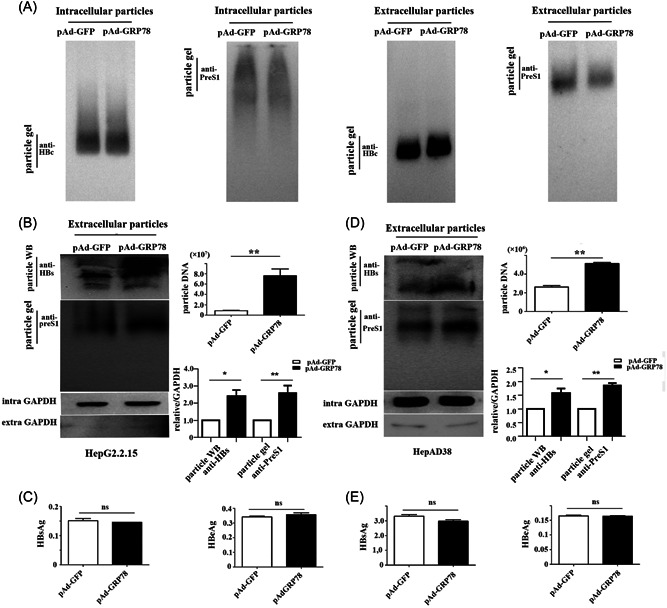
GRP78 overexpression promotes HBV virion release. (A) Particle gel assay detecting HBV particles using anti‐preS1 and anti‐HBcAg antibodies in the culture supernatant and cytoplasm of HepG2.2.15 cells infected with pAd‐GRP78. (B) HBV DNA content in HepG2.2.15 extracellular particles. (C) HBeAg and HBsAg in the culture supernatant of Ad‐GRP78‐treated HepG2.2.15 cells (*p* > 0.05 [*n* = 3]). (D) HBV DNA content in HepAD38 cells. (E) Relative levels of HBeAg and HBsAg in the culture supernatant of Ad‐GRP78‐treated HepAD38 cells (*p* > 0.05 [*n* = 3]). GRP78, glucose‐regulated protein 78; HBcAg, hepatitis B core antigen; HBeAg, hepatitis B e‐antigen; HBsAg, hepatitis B surface antigen; HBV, hepatitis B virus.

To verify these findings, we detected both the core particle and enveloped particle as intracellular and extracellular components via particle gel assays. The results showed that GRP78 overexpression had negligible effects on the core protein content and virion abundance in the cytoplasm, as well as core protein abundance in the culture supernatant of HepG2.2.15 cells. By contrast, GRP78 significantly increased the abundance of virions in the culture supernatant relative to that in the control (Figure 2A). Moreover, western blot and qPCR results revealed significantly higher viral DNA content and envelope protein abundance in the supernatant of pAd‐GRP78‐treated HepG2.2.15 cells relative that in cells infected with pAd‐GFP (Figure 2B). Furthermore, we detected a significantly higher virion content in the supernatant of Ad‐GRP78‐treated HepAD38 cells relative to those treated with Ad‐GFP (Figure 2D). Notably, the abundance of neither hepatitis B surface antigen (HBsAg) nor hepatitis B e‐antigen (HBeAg) was significantly altered in the culture supernatant of the two GRP78‐overexpressing cell lines (Figure 2C,E). These results suggested that GRP78 overexpression might promote the release of HBV virions while having negligible effects on free antigen (HBsAg and HBeAg) and core particle abundance.

### 3.3 GRP78 knockdown suppresses HBV virion release

**Figure 3:**
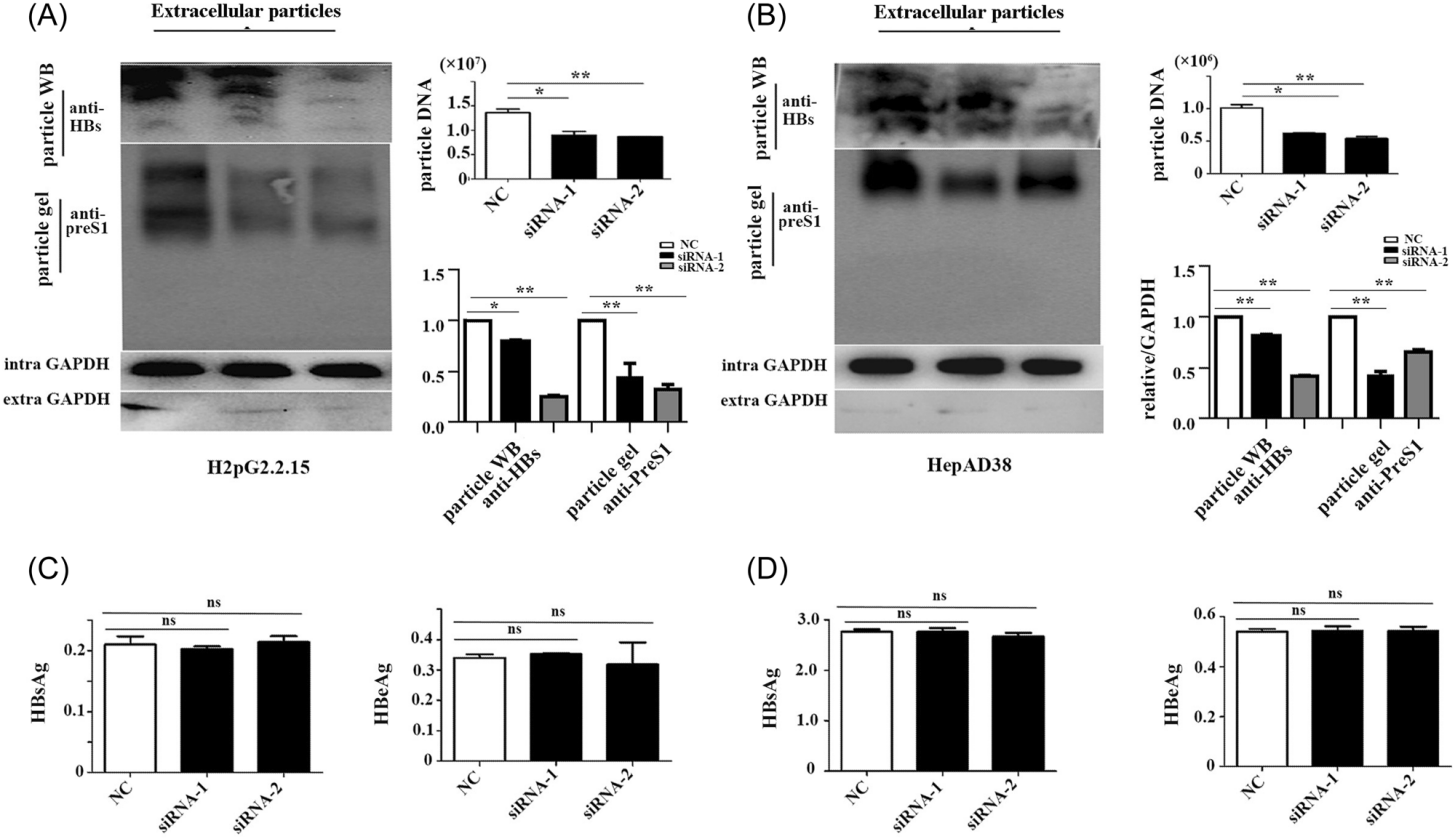
GRP78 knockdown disrupts the release of the HBV virion. (A) HBV DNA content in HepG2.2.15 particles and abundance of extracellular HBV particles and the HBsAg envelope protein. (B) Relative levels of HBeAg and HBsAg in the supernatant of cultured HepG2.2.15 cells treated with siRNA (*p* > 0.05 [*n* = 3]). (C) HBV DNA and HBV virions released from HepAD38 cells. (D) Relative levels of HBeAg and HBsAg in the culture supernatant of HepAD38 cells treated with siRNA (*p* > 0.05 [*n* = 3]). GAPDH expression was used as a loading control. GAPDH, glyceraldehyde 3‐phosphate dehydrogenase; GRP78, glucose‐regulated protein 78; HBeAg, hepatitis B e‐antigen; HBsAg, hepatitis B surface antigen; HBV, hepatitis B virus; siRNA, small‐interfering RNA.

We then used small‐interfering (si)RNA to knockdown GRP78 expression in HepG2.2.15 and HepAD38 cells (Supporting Information: Figure S4). Particle gel assays revealed significant reductions in the abundance of HBV virions in the culture supernatant of siRNA‐treated cells relative to levels in those with normal GRP78 expression (Figure 3A). Additionally, we observed decreases in the HBV envelope protein and viral DNA content in the supernatant according to western blot and qPCR analyses (Figure 3A). By contrast, enzyme‐linked immunosorbent assays indicated that GRP78 knockdown did not significantly affect HBsAg or HBeAg abundance in the HepG2.2.15 supernatant (Figure 3C), with similar results observed in HepAD38 cells (Figure 3B,D). These results suggested that GRP78 positively promoted the secretion of HBV‐enveloped particles.

### 3.4 GRP78 promotes HBV virion release in vivo

**Figure 4:**
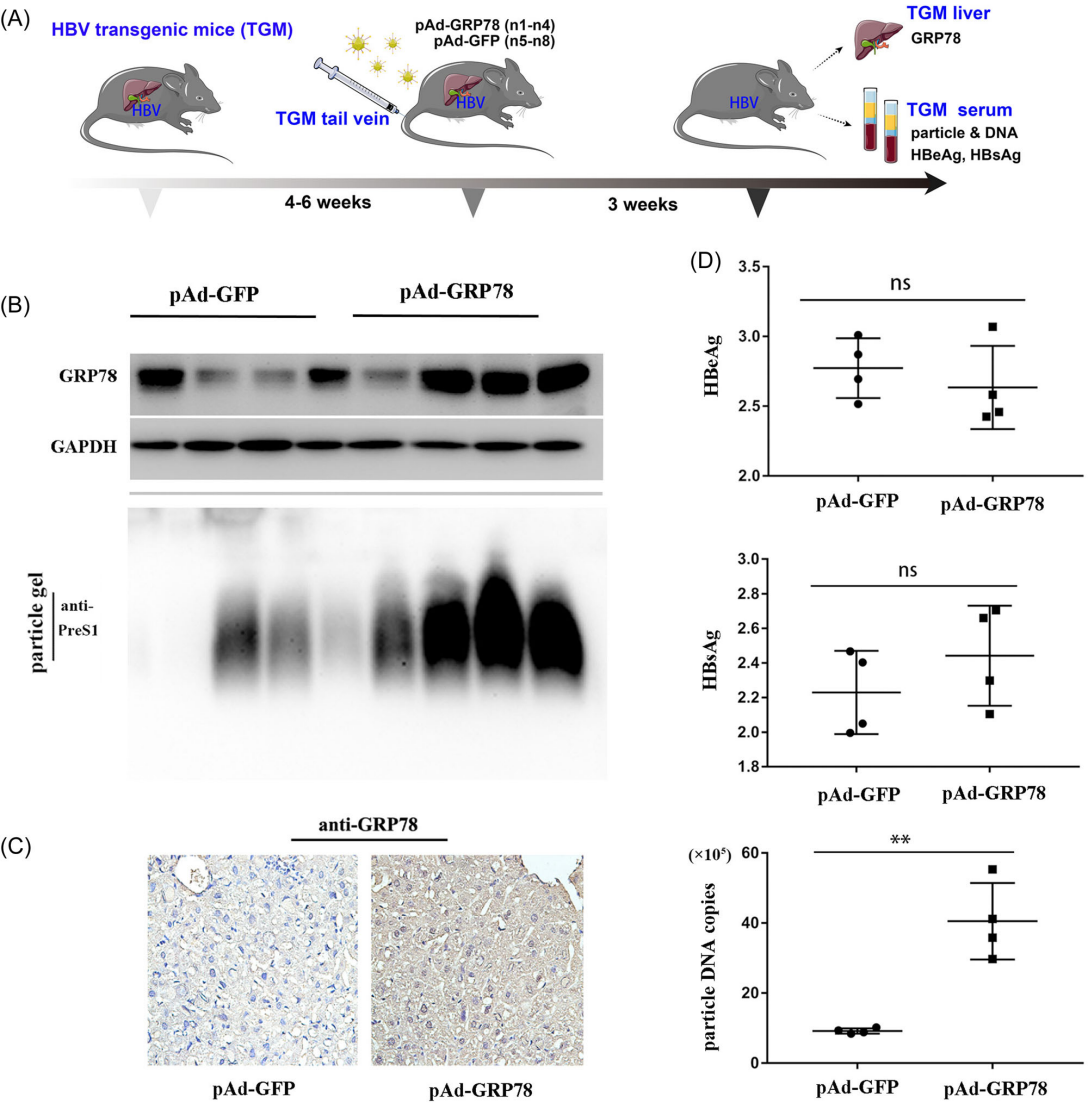
GRP78 overexpression in HBV TgM increases virion release. (A) Study design. (B) GRP78 expression in liver tissue lysates, with GAPDH used as the internal control. Abundance of serum HBV virions. (C) Pathological liver characteristics of TgM treated with Ad‐GRP78 or Ad‐GFP. (D) Serum levels of HBeAg, HBsAg, and HBV DNA content (**p < 0.01 [*n* = 4]). GAPDH, glyceraldehyde 3‐phosphate dehydrogenase; GFP, green fluorescent protein; GRP78, glucose‐regulated protein 78; HBeAg, hepatitis B e‐antigen; HBsAg, hepatitis B surface antigen; HBV, hepatitis B virus; TgM, transgenic mice.

To investigate whether HBV particles are affected by GRP78 in vivo, we randomly divided eight HBV TgM into control and experimental groups treated with pAd‐GRP78 or pAd‐GFP via tail‐vein injection (Figure 4A), followed by extraction of serum and liver tissues after 3 weeks. Western blot and immunohistochemical analyses confirmed GRP78 overexpression in the liver tissues of pAd‐GRP78‐treated HBV TgM (Figure 4B,C). Additionally, particle gel assays and qPCR analysis revealed that GRP78 significantly upregulated the virion abundance and viral DNA content in serum from pAd‐GRP78‐treated HBV TgM (Figure 4B,D). Notably, we observed negligible differences in serum HBsAg and HBeAg levels between groups (*p* > 0.05). These findings were consistent with the in vitro results, further supporting that GRP78 overexpression can promote HBV virion release.

### 3.5 The GRP78 SBD and NBD synergistically participate in HBV virion release

**Figure 5:**
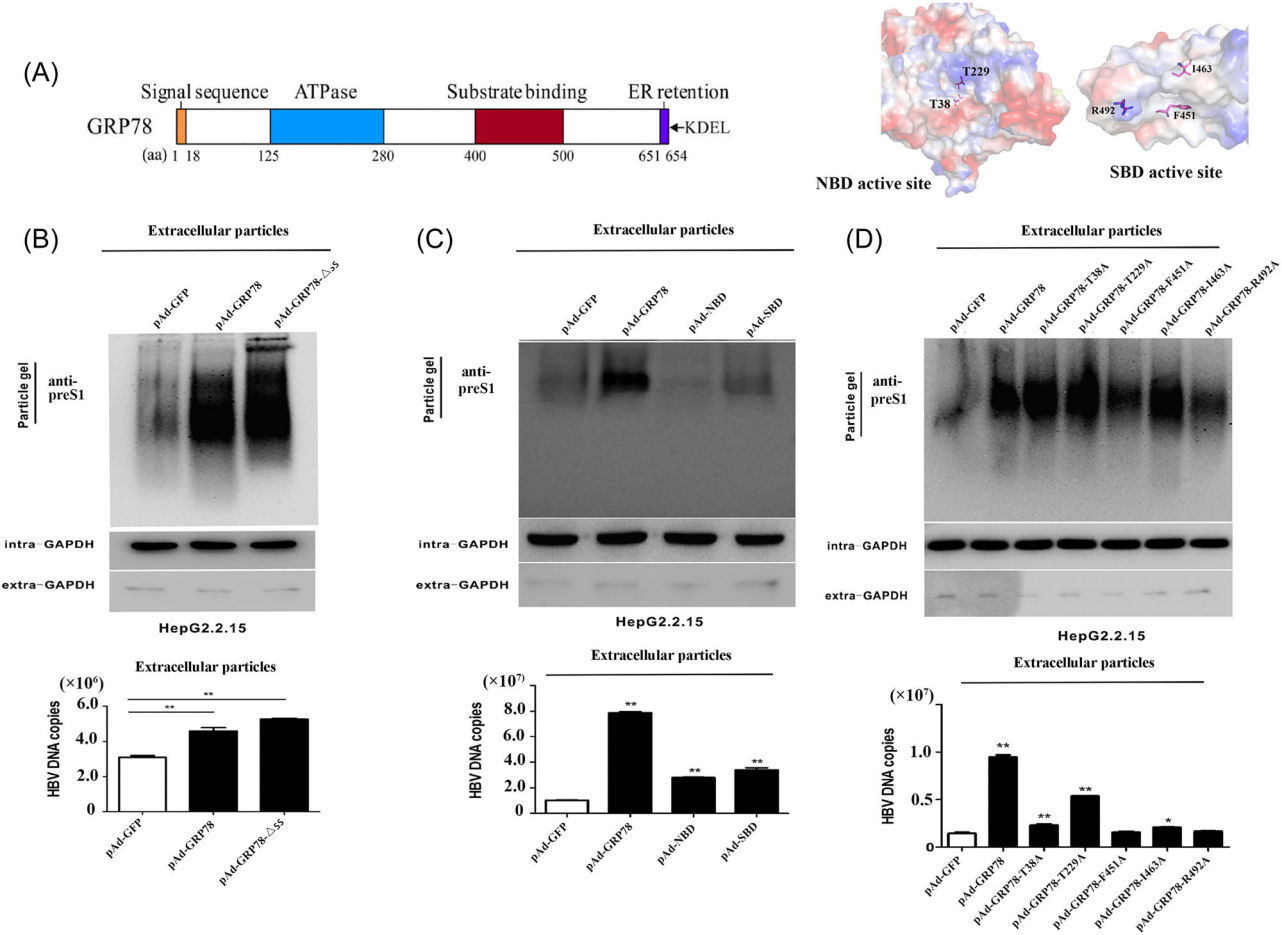
The SBD and NBD of GRP78 synergistically participate in the release of the HBV virion. (A) Schematic of the GRP78 sequence and domains. The signal sequence, ATPase domain (NBD), and SBD are highlighted in yellow, blue, and red boxes, respectively. (B) Abundance of HBV virions released from HepG2.2.15 cells treated with pAd‐GFP, pAd‐GRP78, and pAd‐GFP‐Δss, respectively (**p < 0.01 [*n* = 3]). (C) HBV virions in the culture supernatant of HepG2.2.15 cells infected with pAd‐GFP, pAd‐GRP78, pAd‐NBD, and pAd‐SBD, respectively (**p < 0.01 [*n* = 3]). (D) HBV virio*n*s released from HepG2.2.15 cells treated with Ad‐GFP, Ad‐GRP78, and five mutants (Ad‐GRP78‐T38A, Ad‐GRP78‐T229A, Ad‐GRP78‐F451A, Ad‐GRP78‐I463A, and Ad‐GRP78‐R492A), respectively (**p < 0.01 [*n* = 3]). GFP, green fluorescent protein; GRP78, glucose‐regulated protein 78; HBV, hepatitis B virus; NBD, nucleotide‐binding domain; SDB, substrate‐binding domain.

In *Homo sapiens*, GRP78 functions as a molecular chaperone protein with a signal peptide and two functional domains (SBD and NBD) (Figure 5A).^14^, ^30^ Therefore, we constructed three adenoviruses with truncated genes (pAd‐GRP78‐Δss, pAd‐SBD, and pAd‐NBD) (Supporting Information: Figure S6).^14^, ^30^ Subsequent particle gel assays and qPCR analysis revealed that virion abundance and viral DNA content increased significantly in the culture supernatant of HepG2.2.15 cells treated with pAd‐GRP78 and pAd‐GRP78‐Δss, respectively (Figure 5B), whereas the GRP78 NBD and SBD showed minimal effects on the abundance of enveloped particles and viral DNA copies in culture supernatant (Figure 5C). Additionally, we constructed five adenoviruses overexpressing active site mutants (including NBD mutants [T38A and T229A] and SBD mutants [F451A, I463A, and R492A]) (Supporting Information: Figure S6), with assessments of their effects revealing an attenuated capacity to promote the release of viral particles and viral DNA as compared with WT GRP78 (Figure 5D). Specifically, the T38A, F451A, I463A, and R492A variants displayed almost no ability to promote enveloped‐particle release, whereas the T229A mutant retained 50% of its effect (Figure 5D), which may have resulted from the compensatory role of endogenously expressed GRP78. Collectively, these data suggested that the SBD and NBD appeared to act synergistically to increase the release of HBV virions, suggesting that GRP78 inhibitors might effectively inhibit the release of intact viral particles.

### 3.6 preS1 peptides interfere with GRP78‐induced increases in HBV virion release

**Figure 6:**
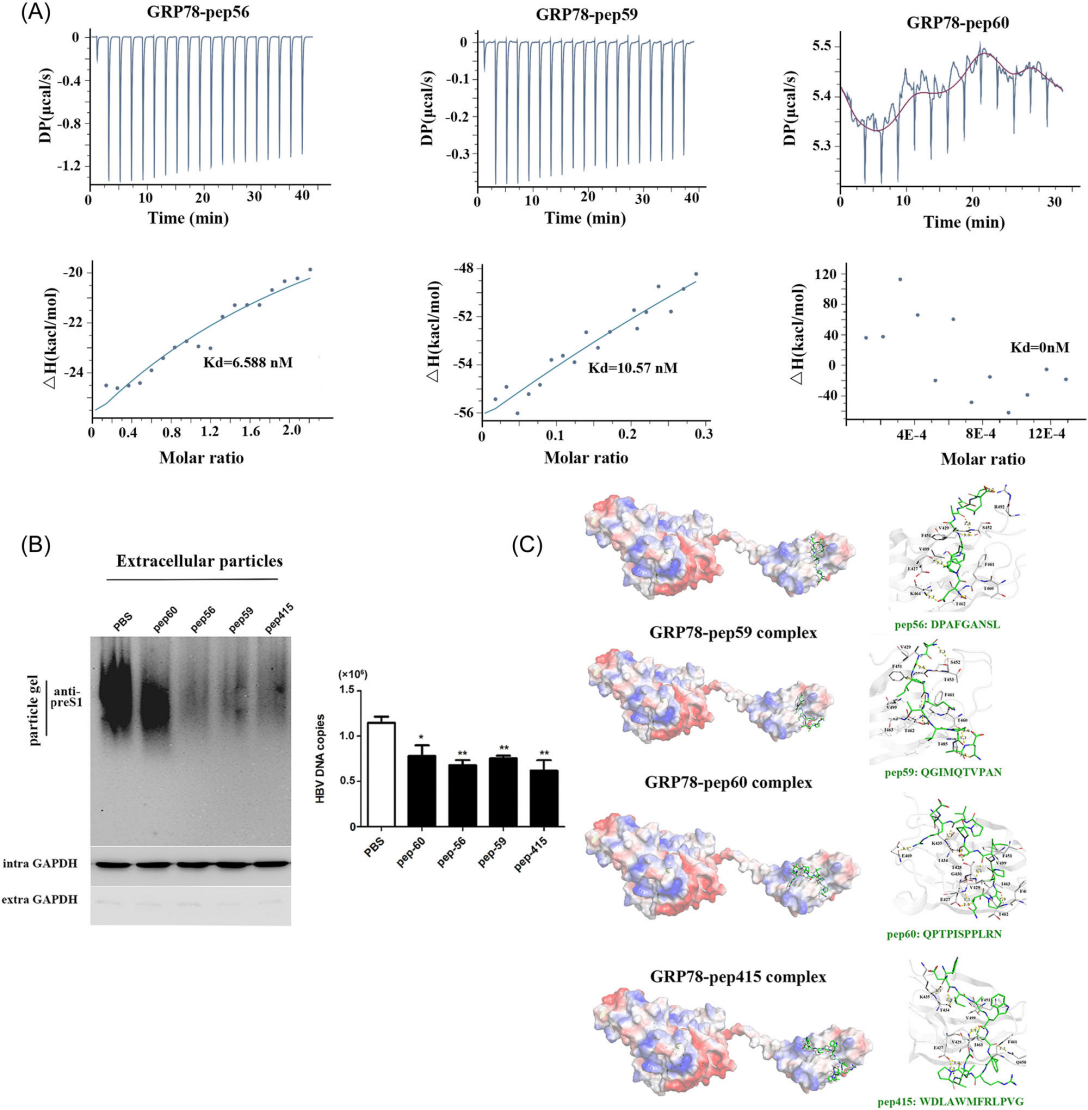
The preS1 peptide inhibits HBV virion release. (A, B) Interaction between recombinant GRP78 and peptides (pep56, pep59, and pep60) according to ITC analysis. (C) Abundance of HBV virions released from HepG2.2.15 cells treated with pep56, pep59, pep60, and pep415 (**p < 0.01 [*n* = 3]). (D) The three‐dimensional model of the GRP78–peptide homodimer complex (pepp56, pep59, pep60, and pep415) using AutoDock Vina (PDB: 6HAB). Cartoon and electrostatic potential surface representation of the overall structure of the complex. A saturated red color indicates Ø <− 10 kiloteslas/e, and a saturated blue indicates Ø > 10 kiloteslas/e (*T* = 293 K). The hydrogen‐bond interaction network between GRP78 and the peptides is shown in Supporting Information: Table S2. GRP78, glucose‐regulated protein 78; HBV, hepatitis B virus; ITC, isothermal titration calorimetry; PDB, Protein Data Bank.

According to the structural and functional features of GRP78, the SBD binds to hydrophobic peptides, whereas the NBD promotes interactions by hydrolyzing ATP to ADP. Several inhibitors, including catechins, epigallocatechin gallate, honokiol, and aspirin, can restrict this ATPase activity, whereas the peptide WDLAWMFRLPVG (pep145) interacts directly with the SBD domain.^33^ Therefore, to investigate whether the effect of a molecular chaperone on HBV is based on the interaction between GRP78 and preS1, we designed three hydrophobic peptides (19‐DPAFGANSL‐29 [pep56], 70‐QGIMQTVPAN‐80 [pep59], and 91‐QPTPISPPLRN‐102 [pep60]) and performed hydrophobicity analysis of preS1 to determine blockage of the preS1–GRP78 interaction. MTS assays showed that the constructed peptides demonstrated minimal levels of cytotoxicity against HepG2.2.15 cells (data not shown). ITC assay results revealed that pep56 and pep59 interacted with recombinant GRP78 with high affinity (Kd = 6.588 × 10^−9^ M and 10.57 × 10^−9^ M, respectively), whereas a weak interaction was observed between pep60 and GRP78 (data not shown) (Figure 6A,B). Interestingly, similar to pep145, both pep56 and pep59 inhibited the ability of GRP78 to enhance enveloped‐particle release and viral DNA content in GRP78‐overexpressing HepG2.2.15 cells, whereas pep60 exhibited minimal effects (Figure 6C). Furthermore, molecular docking of these peptide–GRP78 complexes (Supporting Information: Table S2) revealed binding scores for pep415, pep56, pep59, and pep60 of −12.2496, −10.0518, −11.1134, and −11.3549 kcal/mol, respectively. Specifically, we found that hydrophilic and hydrogen‐bond interactions were overcome by the strong hydrophobic interactions between specific GRP78 residues (F451, V499, I463, V429, and F461) and hydrophobic residues located in the middle of the respective peptides (e.g., Phe4 [pep56], Ile3 [pep59], and Met6 [pep415]). However, we observed less of a hydrophobic interaction between GRP78 and pep60, which explained the poor inhibitory effect on virion release.

### 3.7 Screening peptides by phage display to inhibit HBV‐enveloped particle secretion

**Figure 7:**
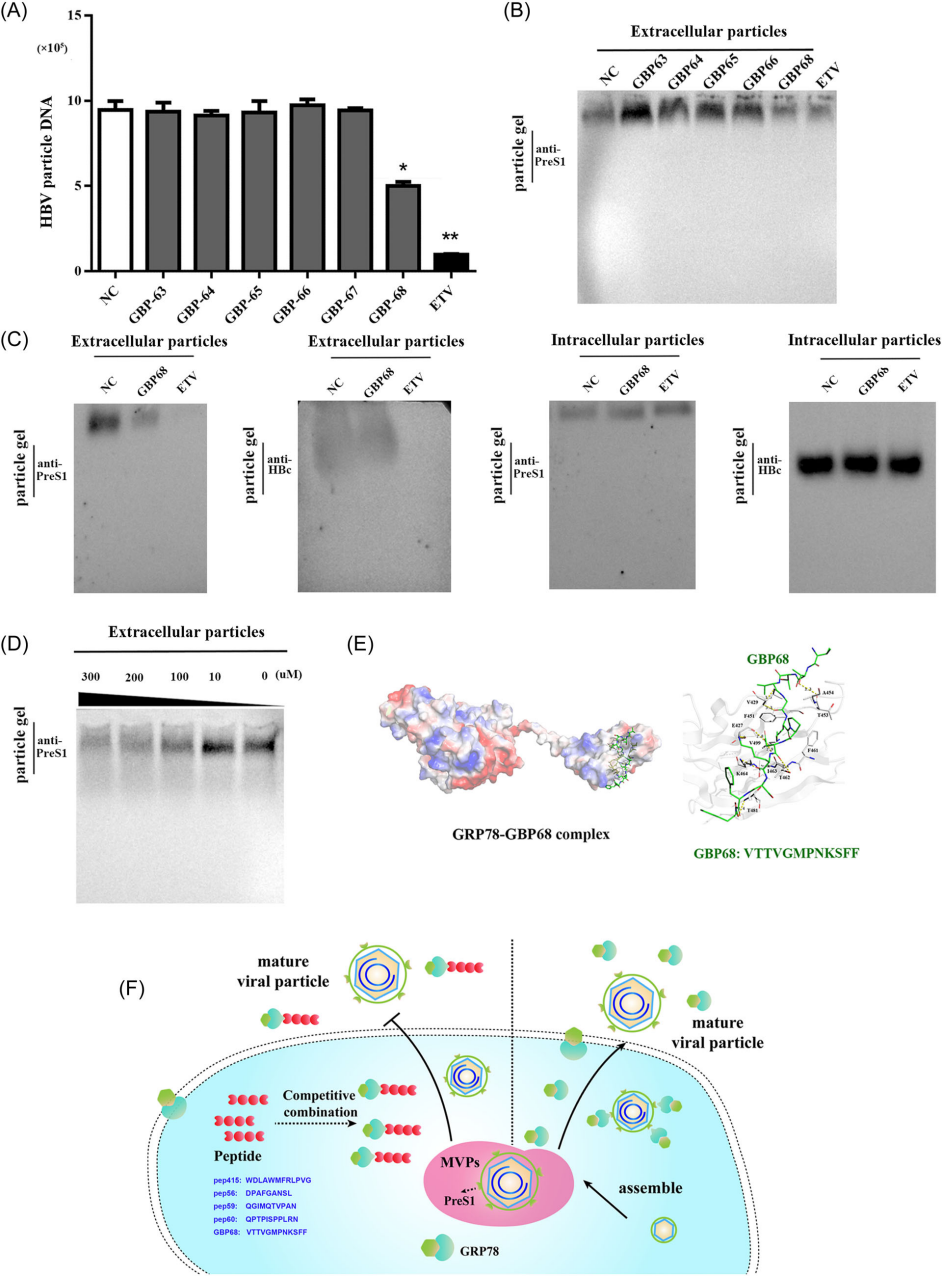
Peptides screened by phage‐display assays interfere with HBV virion release. (A) DNA copies of HBV particles from HepG2.2.15 cells treated with peptides (GBP‐63, −64, −65, −66, −67, and −68) according to qPCR analysis. (*p < 0.05, **p < 0.01 [*n* = 3]). (B) HBV‐enveloped particles from HepG2.2.15 cells treated with six peptides (GBP‐63, −64, −65, −66, −67, and −68), respectively, were determined by particle gel assays using the anti‐preS1 antibody. (C) Detection of HBV core particles using the anti‐HBcAg antibody and enveloped particles using the anti‐preS1 antibody in the cytoplasm of HepG2.2.15 cells and culture supernatant following treatment with GBP‐68 or entecavir. (D) HBV‐enveloped particles released from HepG2.2.15 cells treated with different concentrations of GBP‐68 according to particle gel assays using the anti‐preS1 antibody. (E) The modeled structure of the GRP78–GBP‐68 complex (GRP78 structure was derived from PDB: 6HAB) generated using AutoDock Vina. Cartoon and electrostatic potential surface representation of the overall structure of the complex. A saturated red color indicates Ø <− 10 kiloteslas/e, and a saturated blue color indicates Ø > 10 kiloteslas/e (*T* = 293 K). The residues involved in hydrogen bonding between GRP78 and the peptides are shown in Supporting Information: Table S2. (F) The GRP78 complex shown here regulates the secretion of HBV‐enveloped particles. GRP78 promotes intact HBV‐enveloped particle release by directly interacting with preS1 on the viral envelope; however, competitive interaction of peptides with GRP78 can inhibit this activity by reducing the interaction between GRP78 and preS1/HBV particles. GRP78, glucose‐regulated protein 78; HBcAg, hepatitis B core antigen; HBV, hepatitis B virus; PDB, Protein Data Bank.

Phage display is a powerful method for screening binding peptides from a highly diverse combination of libraries.^33^ To design GRP78 inhibitors, we employed a 12‐peptide phage library to screen candidate peptides. Following three rounds of biopanning, we selected 144 clones for Sanger sequencing (data not shown), with eight peptides showing multiple frequencies (>2) (GBP‐63 [pep7], −64 [pep11], −65 [pep16], −66 [pep19], −67 [pep32], and −68 [pep51]). Among them, we were unable to synthesize pep9 and pep46 due to their level of hydrophobicity (Supporting Information: Table S3); however, synthesis of the remaining six peptides resulted in low cytotoxicity in HepH2.2.15 cells (Supporting Information: Figure S7). Excluding GBP‐67, the remaining five peptides interacted with recombinant GRP78 with high affinity according to ITC analysis (Supporting Information: Table S4). GBP‐68 exerted a significantly stronger inhibitory effect on HBV‐enveloped particle release and viral DNA content in the supernatant of HepG2.2.15 cells as compared with GBP‐63, −64, −65, and −66 (*p* < 0.05) (Figure 7A,B). Furthermore, GBP‐68 inhibited enveloped‐particle release from HepG2.2.15 and HepAD38 cells in a dose‐dependent manner but exerted negligible effects on the abundance of cytoplasmic viral particles and core particle release (Figure 7C,D and Supporting Information: Figure S8). Moreover, molecular‐docking studies revealed that GBP‐68 generated nine hydrogen bonds (<3.50 Å) with GRP78, whereas we observed the key residue (Met6) oriented in a hydrophobic pocket formed by Phe451, Phe461, Val499, and Val511 (Figure 7E), which promoted the stable hydrophobic interaction between GBP‐68 and GRP78. Conversely, no hydrogen bonds were formed between GRP78 and GBP‐63, −65, −66, and −67, whereas six hydrogen bonds were formed with GBP‐64 (Supporting Information: Figure S9). These findings further demonstrated that interactions between peptides and GRP78 could alter HBV‐enveloped particle secretion (Figure 7F and Supporting Information: Figure S10).

## 4 DISCUSSION

The release of HBV‐enveloped particles from infected cells is required for persistent chronic infection; however, the precise regulatory mechanism underlying this release process remains unclear. In this study, we found that GRP78 interacted specifically with HBV particles via the viral envelope protein preS1, and that HBV upregulated GRP78 expression correspondingly. Furthermore, we showed that hydrophobic peptides interacted with GRP78 with high affinity, thereby altering GRP78‐mediated secretion of HBV‐enveloped particles.

We showed that GRP78 positively regulated the release of HBV virions while having negligible effects on the release of core proteins or free viral antigens, including HBsAg and HBeAg. GRP78 is a secretory chaperone with signal peptides that can aid the secretion of various molecules, and preS1, located in the outer membrane of the viral particle, can bind to GRP78 with high affinity, thus facilitating the subsequent release of the enveloped particle. Conversely, HBsAg, HBeAg, and viral core proteins do not directly interact with GRP78; therefore, their release is not regulated by GRP78. The release of HBV virions causes an increase in certain extracellular viral biomarkers, including viral DNA, hepatitis B core antigen, and HBsAg, whereas virions are only a small fraction of the markers excreted by the virus. As a result, their variation does not cause significant changes in serum levels of these markers.^6^, ^7^

Virion morphogenesis involves interactions between HBc, envelope proteins (HBsAg), and host factors, such as components of the ESCRT machinery. Vps4, a host factor involved in the sorting of cellular vacuolar proteins, is associated with ESCRT‐mediated membrane dynamics. HBV replication and core particle secretion are significantly inhibited by Vps4.^34^, ^35^ ESCRT can mediate the export of several viruses, with >15 ESCRT proteins, including charged multivesicular body protein (CHMP)3, CHMP4B, CHMP4C, ELL‐associated protein (EAP)20, EAP30, and EAP45, required for HBV replication and release.^35^, ^36^ Host cellular kinases, including protein kinase C, SRSF protein kinase 1/2, cyclin‐dependent kinase 2, polo‐like kinase 1, and protein phosphatase 1, can regulate the phosphorylation of HBV capsids, thereby inhibiting viral particle formation and promoting intracellular capsid accumulation.^11^, ^12^, ^13^ Furthermore, bone marrow stromal cell antigen‐2/tetherin, an IFN‐inducible antiviral cellular protein can restrict HBV particle release in HepG2 cells, although this effect is not observed in HuH‐7 cells.^37^ Although these host factors reportedly affect HBV‐capsid‐particle release, there is little evidence demonstrating whether they directly interact with preS1 to regulate intact particle release. In the present study, we found that GRP78 interacted directly with preS1, thus positively regulating the release of HBV‐enveloped particles. As a key point in the HBV life cycle, secretion of the enveloped particle may require several host molecules for collaborative regulation of GRP78, especially host proteins that interact with GRP78. These findings along with those of previous studies suggest a model for HBV‐enveloped particle release as being positively regulated by the human host‐shock protein GRP78 (Figure 7F and Supporting Information: S10). Although additional biochemical and structural studies are required to confirm the predicted interactions, the present results using in vitro and in vivo models strongly support this hypothesis. When mature nucleocapsids are further packed as intact viral particles by envelope proteins, the preS1 protein appears in the outermost layer, where it can interact with host proteins. Consequently, certain host proteins in the human liver could directly interact with preS1, thus playing a key role in HBV release. The γ2‐adaptin HEAD domain (N‐terminal) can bind to the HBV nucleocapsid and mediate core particle export by host ESCRT machinery, whereas its EAR domain (C‐terminal) interacts with preS1 and participates in HBV replication.^38^ NTCP specifically interacts with the preS1 region of the HBV L protein and serves as an entry receptor for HBV infection.^26^, ^39^ Although GRP78 can interact with preS1 and function as an intracellular antiviral factor by inhibiting HBV infection, the mechanism underlying this antiviral effect remains elusive. Furthermore, the present study revealed that GRP78 specifically interacts with the preS1 region while exerting a minimal effect on HBV entry into human liver cells. Additionally, GRP78 promoted the secretion of HBV‐enveloped particles in the culture supernatant and serum of TgM by enhancing viral concentration in the cytoplasm. These results suggest the ability of GRP78 to positively regulate viral enveloped‐particle release both in vitro and in vivo. Consequently, GRP78 might represent a target for antiviral drugs to inhibit excretion of HBV‐enveloped particles and thereby inhibit propagation of the viral life cycle.

We further evaluated the hypothesis that peptide interaction with GRP78 would block or interfere with its binding to the viral envelope protein, thus inhibiting enveloped‐particle release. We subsequently identified three preS1 peptides that directly interacted with GRP78 and effectively interfered with secretion of a viral enveloped particle. These results identified the ability of GRP78 to bind peptides with different sequences; however, only some of these peptides were able to interfere with the release of HBV‐enveloped particles, which could be related to the competitive interaction of peptides with formation of the preS1–GRP78 complex.

As a molecular chaperone, GRP78 plays an essential role in the function of viral envelope proteins, including those of Sindbis virus, HCV, vesicular stomatitis virus, and influenza A virus. GRP78 functions as an attachment factor for binding to the MERS‐CoV spike protein and enhances viral entry into host cells.^18^ Importantly, data derived primarily from the analysis of other coronaviruses provide sufficient evidence to support the putative role of CD147 and GRP78 as entry receptors for SARS‐CoV‐2. Specifically, the SARS‐CoV‐2 spike protein can stably interact with the GRP78, implying that it might be involved in SARS‐CoV‐2 entry.^40^ Additionally, the viral cloud of coxsackievirus A9 binds to the host receptor GRP78 and the integrin αvβ3 and then uses major histocompatibility complex‐I for entry into host cells.^41^ Moreover, GRP78 functions as a receptor for JEV by directly interacting with envelope domain III.^21^ GRP78 interacts with the envelope and has been identified as a receptor for dengue virus serotype 2.^42^ Similarly, GRP78 interacts with the HBV envelope protein, whereas it does not serve as a receptor to aid HBV entry into host cells. We demonstrated that GRP78 is involved in the secretion of the enveloped particle, thereby offering a new perspective to clarify the role of GRP78 in the viral life cycle.

We determined that GRP78 directly interacted with preS1 and positively regulated the release of HBV‐encapsulated particles, and that peptides inhibited the secretion of viral particles by disrupting the preS1–GRP78 interaction. These findings provide a basis for a more in‐depth analysis of HBV‐enveloped‐particle excretion, as GRP78 may function as a novel target for designing antiviral therapeutics to inhibit excretion of the viral particle.

## AUTHOR CONTRIBUTIONS

**Yueyuan Shi**: Conceptualization; investigation; resources; data curation; writing – original draft preparation. **Xin Jin**: Conceptualization; investigation; resources; data curation. **Deqiang Wang**: Conceptualization; data curation; writing – review and editing; supervision; project administration; funding acquisition. **Shuang Wu**: Methodology; investigation; resources. **Junye Liu**: Methodology; investigation; resources. **Yuan Yang**: Methodology; investigation. Xiang Zhang: Investigation. Jie Wei: Resources. **Hua Zhou**: Resources. **Hongpeng Zhang**: Resources. **Miao Luo**: Resources. **Xuefei Cai**: Resources. **Ailong Huang**: Writing – review and editing. supervision; project administration; funding acquisition. All authors have read and agreed to the published version of the manuscript.

## CONFLICT OF INTEREST

The authors declare no conflict of interest.

## ETHICS STATEMENT

The study was approved by the Clinical Research Ethics Committee of Chongqing Medical University in January 2021 and complied with the Declaration of Helsinki.

## Supporting information

Supporting information.

## Data Availability

The data presented and supporting information in this study are available on request from the corresponding author.
