# Supplementary material for: Release of hepatitis B virions is positively regulated by glucose‐regulated protein 78 through direct interaction with preS1
Source: J Med Virol. 2022 Dec 1;95(1):e28271. doi: 10.1002/jmv.28271 (PMC10107996; doi:10.1002/jmv.28271)

**The [supplementary](C:/Users/cquwa/AppData/Local/youdao/dict/Application/8.10.3.0/resultui/html/index.html#/javascript:;) [materials](C:/Users/cquwa/AppData/Local/youdao/dict/Application/8.10.3.0/resultui/html/index.html#/javascript:;) of “HBV particle secretion is positively regulated by glucose-regulated protein 78 directly interacting with preS1”**

**1. Experiments and materials**

***1.1. Patients***

Table S1 shows the characteristics of the patients in the present study, including 103 women and 68 men with a median age of 55.2 (18–65 years). The study protocol was approved by the Research Ethics Committee of Chongqing Medical University and written informed consent was obtained from each patient.

***1.2. RNA extraction and real-time quantitative PCR***

Total RNA was extracted from cells (or liver tissues from HBV transgenic mice) using TRIzol reagent (Invitrogen, USA). First-strand cDNA was synthesized as previously described. RT-qPCR was performed using a Bio-Rad sequence detection system according to the manufacturer's instructions using a double-stranded DNA-specific SYBR Green Premix Ex TaqTM II Kit (Roche, Switzerland). Experiments were conducted in duplicate in three independent assays. Relative transcriptional fold was calculated as 2-∆∆CT. GAPDH was used as the internal control for normalization.

***1.3. Protein extraction and western blot detection***

Total protein lysates were extracted from hepatoma cells using radioimmunoprecipitation assay buffer. Protein concentrations were measured using the BCA protein quantification kit (Thermo Scientific, United States), and 20–50 µg protein extracts were subjected to SDS-PAGE. Then, the proteins were transferred to a nitrocellulose membrane, blocked with 5% nonfat milk, and incubated with primary antibodies for 1 h at room temperature. After incubation with secondary antibodies against mouse (1:10,000) or rabbit (1: 10,000) for 1 h at 4℃ overnight, the membrane was visualized using the ECL Western Blotting Detection Kit (Bio-Rad, United States).

***1.4. In vitro binding assay of GRP78 and preS1***

According to standard protocols, recombinant GST-preS1 was produced in Escherichia coli BL21 cells and purified using glutathione sepharose 4B (GE Healthcare, United States). Ten micrograms of GST or GST fusion proteins and cell lysates were incubated at 4 °C overnight. Supernatants were collected as input, and the sepharose beads were then extensively washed six times with lysis buffer and eluted.

***1.5. Immunofluorescence assays***

To analyze the colocalization of preS1 and GRP78, we used the preS1 monoclonal antibody and GRP78 monoclonal antibody (Abcam, England). Cells cultured in 48-well plates were washed three times with precooled phosphate-buffered saline, fixed with 4% paraformaldehyde for 10 min, and permeabilized for 10 min at RT with 0.5% Triton X-100. After incubation for 1 h with 3% bovine serum albumin to block nonspecific binding, primary antibodies were added and incubated for 1 h at 37 °C. The bound antibodies were visualized by incubation with secondary antibodies (Alexa Fluor 488 donkey anti-mouse IgG or Alexa Fluor 594 anti-mouse IgG). Images were acquired using a fluorescence microscope.

***1.6. Co-immunoprecipitation***

Total protein lysates were extracted from hepatoma cells using the IP lysate buffer. The lysate was mixed with 40 µL protein G agarose (Millipore, United States) to avoid nonspecific binding for 2 h at 4 °C. The supernatant was incubated with the primary antibody (Sigma) for 4 h at 4 °C. Subsequently, the mixture was incubated with 60 µL protein agarose for 2 h at 4 °C. The agarose was then washed three times with PBST buffer, boiled, and then loaded onto SDS-PAGE gel for analysis.

***1.7. Isothermal titration calorimetry (ITC)***

Affinity constants under equilibrium (Ka) were obtained using a Nano ITC instrument. GRP78 protein solution (300 µL) was titrated with repeated injections of 40 µL solution until saturation at 25 °C. Nano Analyze software was used for the integration of heat signals and nonlinear regression analysis of the data.

***1.8. Enzyme-linked immunosorbent assay (ELISA)***

The expression levels of HBVe antigen (HBeAg) and HBV surface antigen (HBsAg) were measured using an HBeAg/HBsAg ELISA kit (Kehua, China) according to the manufacturer’s instructions. Data were obtained from at least three independent experiments.

***1.9. Quantification and differential expression analysis of transcripts***

RNA was isolated from HepG2.2.15 cells infected with adenovirus (pAd-GRP78 or pAd-GFP) using TRIzol reagent (Invitrogen, The United States) according to the manufacturer’s instructions, followed by purification on a RNeasy column (Qiagen, German) with DNase treatment. The RNA sequencing library was processed using the Ion Total RNA-Seq Kit v2 (Life Technologies, United States). Then, each library preparation was sequenced using the Ion PI™ chip and raw reads were created. The clean sequences were mapped to the reference genome using Misplacing software, and the gene expression level was normalized to the reads per kilobase per million. To identify differentially expressed genes (DEGs), the DEseq package was used to filter the DEGs for the HepG2.2.15-pAdGRP78 and HepG2.2.15-pAd-GFP groups. After statistical analysis, we screened the DEGs with a fold change > 1.5 or fold change < 0.667, and the false discovery rate threshold was < 0.05. Gene Ontology (GO) analysis was performed using the GO Term Finder tool to identify the main function of the DEGs. To identify significantly enriched pathways, pathway annotation was performed using the Kyoto Encyclopedia of Genes and Genomes database. The significantly enriched pathways were computed based on Fisher’s test of the hypergeometric distribution at p < 0.05.

***1.10. Phage display***

The prokaryotic expression and purified GRP78 were diluted to 100 μg/mL in the Tris buffered saline (50 mM Tris, 150 mM NaCl, pH 7.5), and 150 μL of protein was adsorbed onto a polystyrene microtiter plate. It was then coated with GRP78, incubated for 16 h at 4 °C, and blocked (2 h at 37 °C) with Tris buffered saline containing 5 mg/mL bovine serum albumin. Then, a phage library (New England Biolabs, USA) was added. After three rounds of phage panning, a single clone was isolated for DNA sequencing.

***1.11. Microscale thermophoresis (MST)***

The interaction between GRP78 and preS1 was confirmed by microscale thermophoresis (48). Experiments were performed with an MST power of 60%, LED power of 40%, and capillaries with a hydrophobic coating under standard conditions. Purified GRP78-His and preS1-GST (preS1-p1-GST, preS1-p2-GST, and preS1-p3-GST) was buffer exchanged into phosphate-buffered saline buffer (pH 7.4), and its concentration was adjusted to 10 µM using UV absorbance. GRP78-His was fluorescently labeled with NT-647 following the manufacturer’s protocol. The preS1-GST solution was diluted to a concentration range of 1.0 × 10-10 to 1.0 × 10-3 mM, and the preS1-GST fusion protein was incubated with the labeled protein for 10 min in the interaction buffer. The samples were then loaded into Monolith NT.115 Capillaries (NanoTemper Technologies) using 50% IR laser power and an LED excitation source (λ= 470 nm) at room temperature. The Kd values were calculated for the interactions between GRP78-His and preS1-GST using NanoTemper Analysis 1.2.20 software. The measurements were replicated three times.

**Table S1.** GRP78 expression in chronic hepatitis B (CHB) patients (92 cases) and healthy individuals (79 cases)


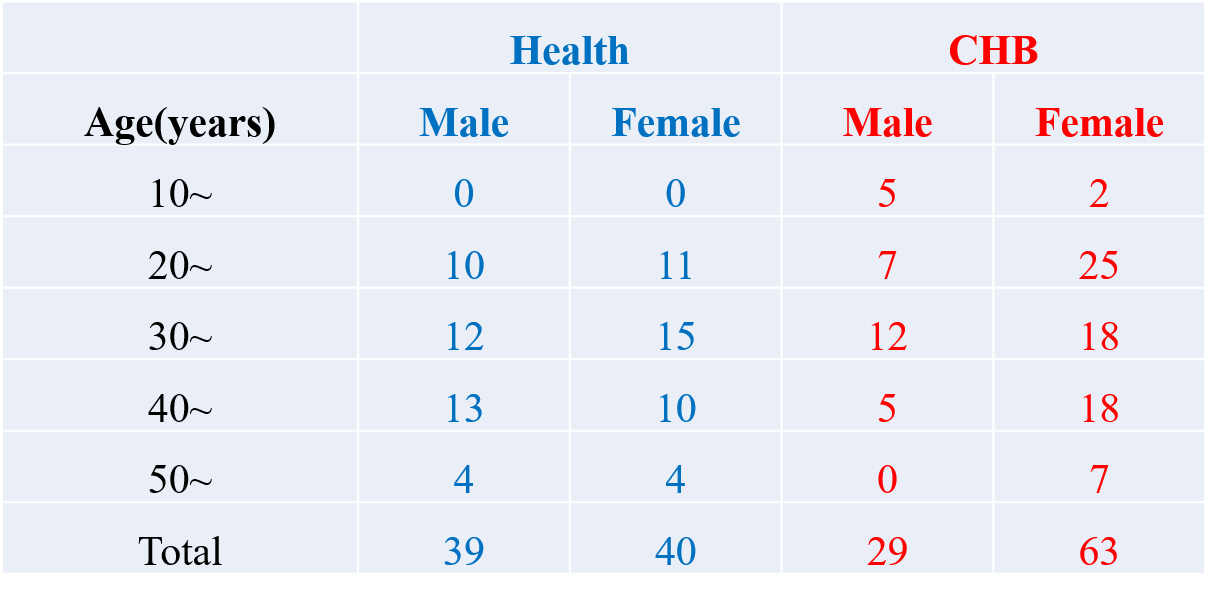


**Table S2.** The docking data of the peptide–GRP78 complex

| **No.** | **Sequence** | **Binding energy (kcal/mol)** | **Hydrogen bonds** | **Residues for hydrogen bonds** | **Hydrophobic contacts** |
| --- | --- | --- | --- | --- | --- |
| **pep415** | WDLAWMFRLPVG | -12.2496 | 6 | T434, K435, E427, E427, V429, Q458 | F451, V499, I463, V429, F461 |
| **pep56** | DPAFGANSL | -10.0518 | 6 | E427, S452, S452, T462,  K464, R492, | F451, V499, I463, V429, F461 |
| **pep59** | QGIMQTVPAN | -11.1134 | 9 | S452, T453, T460, T460,  T460, T462, T462, T462, T485 | F451, V499, I463, V429, F461 |
| **pep60** | QPTPISPPLRN | -11.3549 | 8 | E427, T428, V429, G430,  T434, K435, T462, E469 | F451, V499, I463, V429, F461 |
| **GBP68** | VTTVGMPNKSFF | -12.008 | 9 | E427, E427, V429, V429,  T453, T462, T462, K464, T481 | F451, V499, I463, V429, F461 |

**Table S3.** Summary of peptides with high frequency sequencing from phage display.

| **No.** | **Sequence peptide no.** | **Peptide sequence** | **Frequency**  **(target/total)** |
| --- | --- | --- | --- |
| **GBP-63** | pep7 | FHSHHNRPMKSP | 5/144 |
| **GBP-64** | pep11 | GSWNTFRAQPTI | 2/144 |
| **GBP-65** | pep16 | LTPHKHHKHLHA | 57/144 |
| **GBP-66** | pep19 | MKAHHSQLYPRH | 9/144 |
| **GBP-67** | pep32 | SNIGALQFLPPP | 3/144 |
| **GBP-68** | pep51 | VTTVGMPNKSFF | 2/144 |

**Table S4.** The interaction between synthesized peptides and recombinant GRP78 by ITC assay.

|  | **GBP63** | **GBP64** | **GBP65** | **GBP66** | **GBP67** | **GBP68** |
| --- | --- | --- | --- | --- | --- | --- |
| **N（sites)** | 0.248±3.22 | 2.88±0.501 | 10.0±1.05 | 10.0±2.38 | negative | 5.79±0.738 |
| **KD(mol)** | 21.6e^-6^±35.7e^-6^ | 17.8e^-6^±21.7e^-6^ | 42.8e^-6^±25.2e^-6^ | 56.2e^-6^±70.8e^-6^ | negative | 15.6e^-6^ ±9.80e^-6^ |
| **△H**  **(kcal/mol)** | 8.83±119 | 0.486±0.186 | 2.58±0.647 | 1.55±0.647 | negative | 3.49±0.881 |

**Figure legends**

**Figure S1**. Results of identification of proteins by mass spectrometry.

A. The expression and purification of GST-preS1. HBV preS1 has an important location as a "decode protein" to find host proteins that can interact with it. The preS1-GST fusion plasmid was constructed, and the recombinant protein fixed the bait protein on the glutathione affinity resin for the subsequent pull-down experiment. B. The proteins detected by mass spectrometry from pull-down by GSP-preS1 with HepG2 cell lysis. C, D. MS/MS spectra of peptides for GRP78 and LANCL1. MS/MS spectra of peptide IINEPTAAAIAYGLDK from GRP78.(M+H)^+^ =1,659.1527. MS/MS spectra of peptide AFPNPYADYNK from LANCL1. (M+H) ^+^ = 1,299.2927.


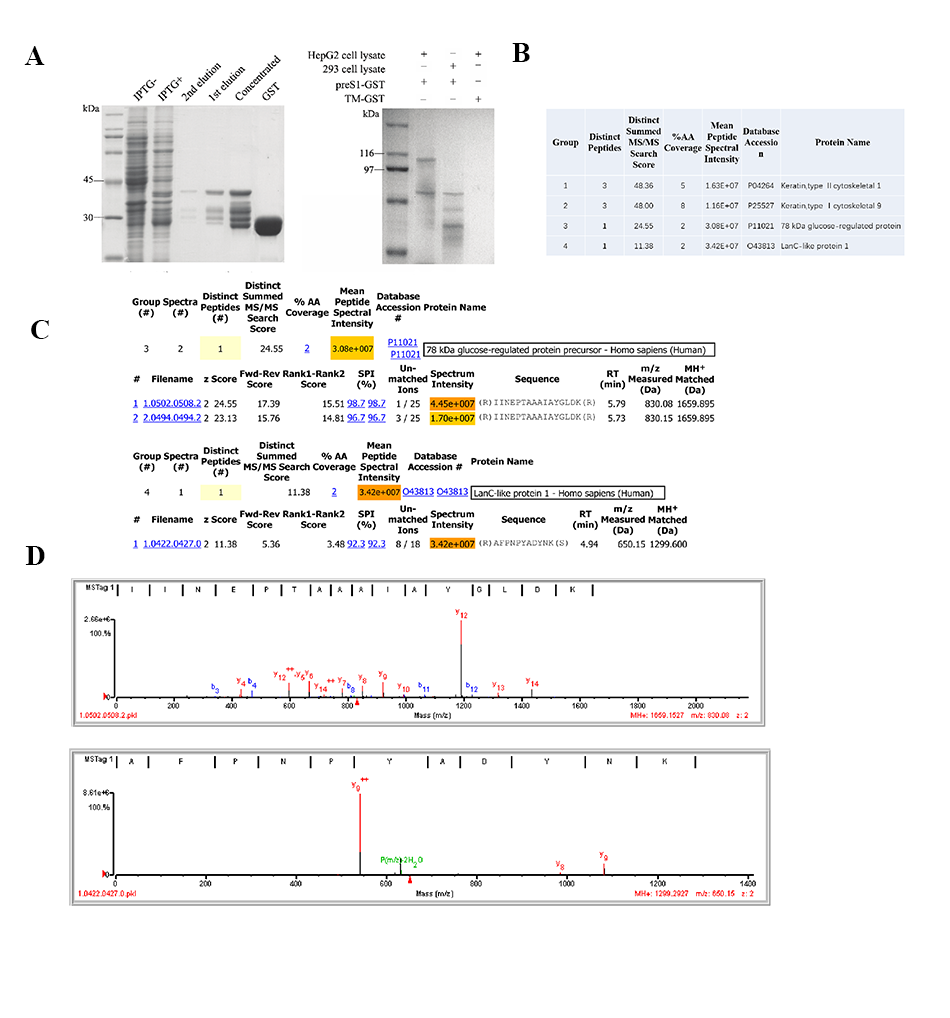


**Figure S2. Interaction between GRP78 and preS1.**

A. The expression and purification of recombinant GRP78 from Escherichia coli BL21. Lane 1, protein marker; lane 2, supernatant; lane 3, flowthrough; lanes 5 and 6, washes with 20 and 40 mM imidazole, respectively; lane 7, elution with 200 mM imidazole. B. The scheme showing three domains of preS1 and three truncated somatic proteins. C. The expression and purification of recombinant GST-preS1 truncated proteins (GST-preS1-p1, GST-preS1-p2, GST-preS1-p3) from E. coli BL21. Lane 1, protein marker; lane 2, supernatant; lane 3, flowthrough; lane 5, washes with phosphate-buffered saline (PBS); lane 7, elution with reduced glutathione in PBS. D. The interaction between GST-preS1 truncated proteins (GST-preS1-p1, GST-preS1-p2, GST-preS1-p3) and recombinant GRP78 by microscale thermophoresis (MST).


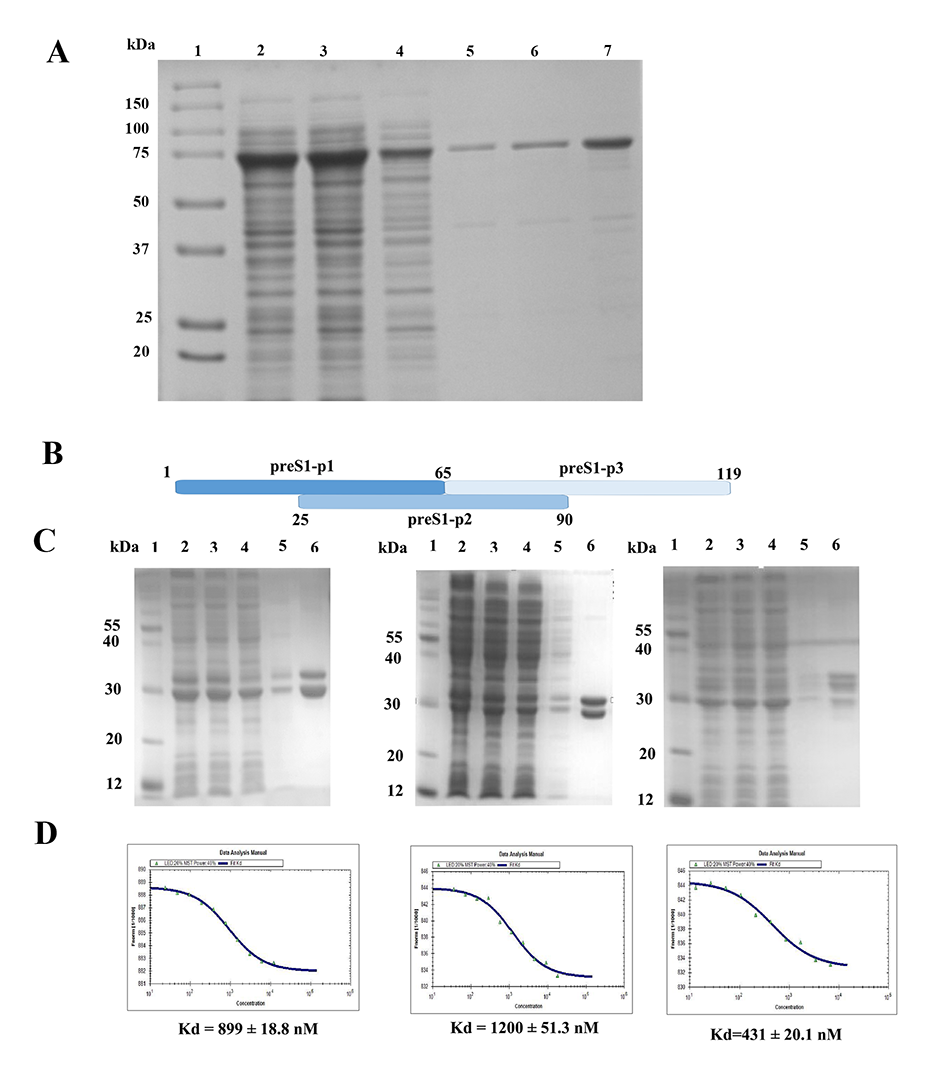


**Figure S3.**

A. Flow chart of HBV affecting GRP78 expression in the HepG2-NTCP cell line.

B. The mRNA expression levels of unfolded protein response (UPR) molecular markers in HepG2 cells treated with HBV plasmids. GRP78, PERK, eIF2α, ATF4, IRE1α, XBP1 and other related proteins were upregulated by overexpression of HBV in HepG2 cells as observed by the detection of UPR molecular markers.


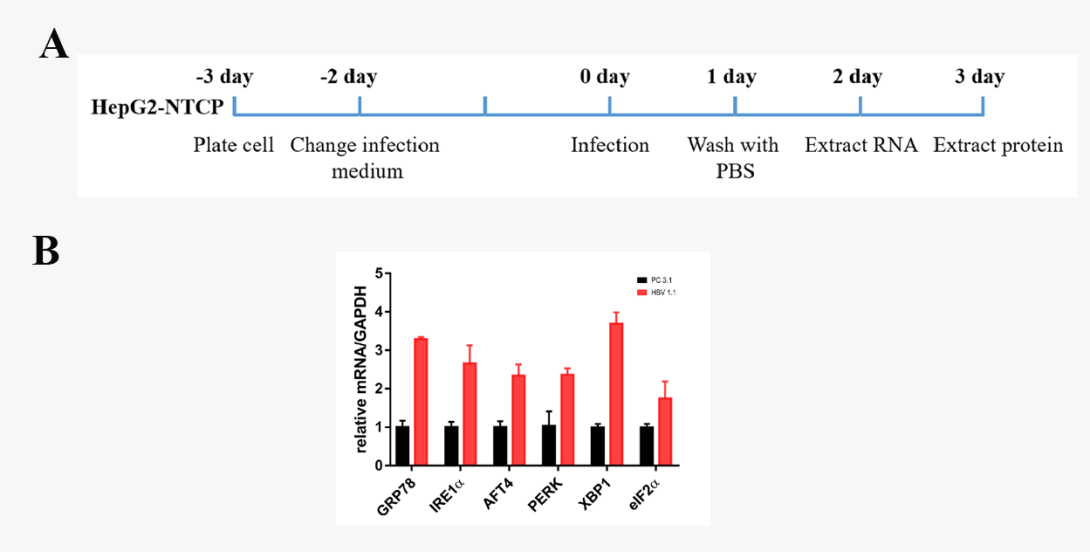


**Figure S4.**

A&B. Verification of the effect of GRP78 overexpression by adenovirus in HepG2.2.15 and HepAD38 cell lines.

C&D. Verification of the effect of GRP78 siRNA in the HepG2.2.15 and HepAD38 cell lines.


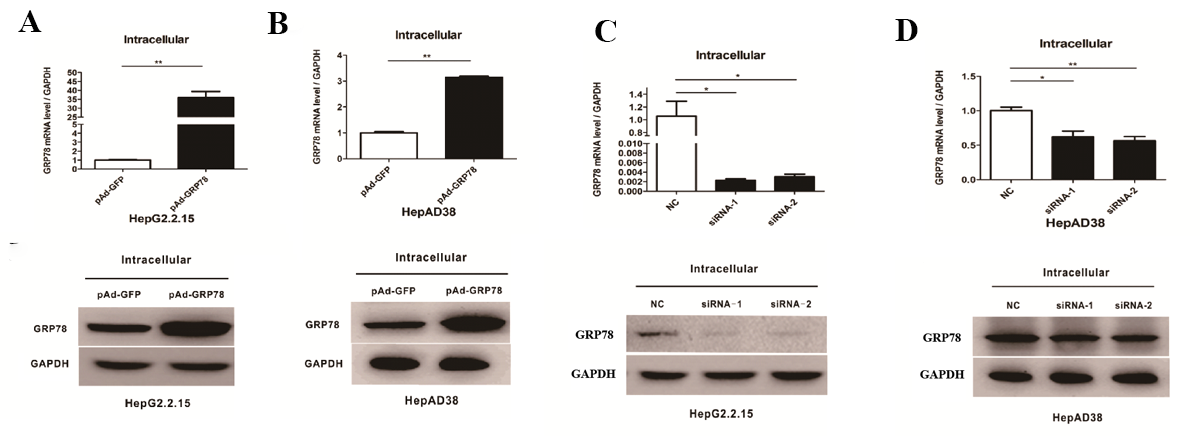


**Figure S5. High-throughput transcriptome sequencing of GRP78 overexpression in the HepG2.2.15 cell line.** A. GRP78 adenovirus was constructed and transferred into HepG2.2.15 cells. B. High-throughput transcriptome (contract number: LBYY-151102-RNA-FDQ-CQ) was commissioned by Liebing Technology (China). The mRNA level of GRP78 was found to be nearly 10-fold higher than that of the control group, which was also a quality control. Horizontal axis represents the number or the range of bases; vertical axis represents the value of mass fraction. Quality scores over 20 suggests that the mapping accuracy is greater than 99%, and these data can be used for subsequent analysis. At the same time, the GC content in the sequence was normally distributed and the data was qualified level. C–E. Bioinformatics analysis of transcriptome sequencing. According to transcriptome sequencing data, the gene transcription level of multiple pathways was improved, including the spliceosome, RNA transport, DNA repair, and protein transport. At the time of HBV replication, GRP78 overexpression caused a significant increase in protein secretion.


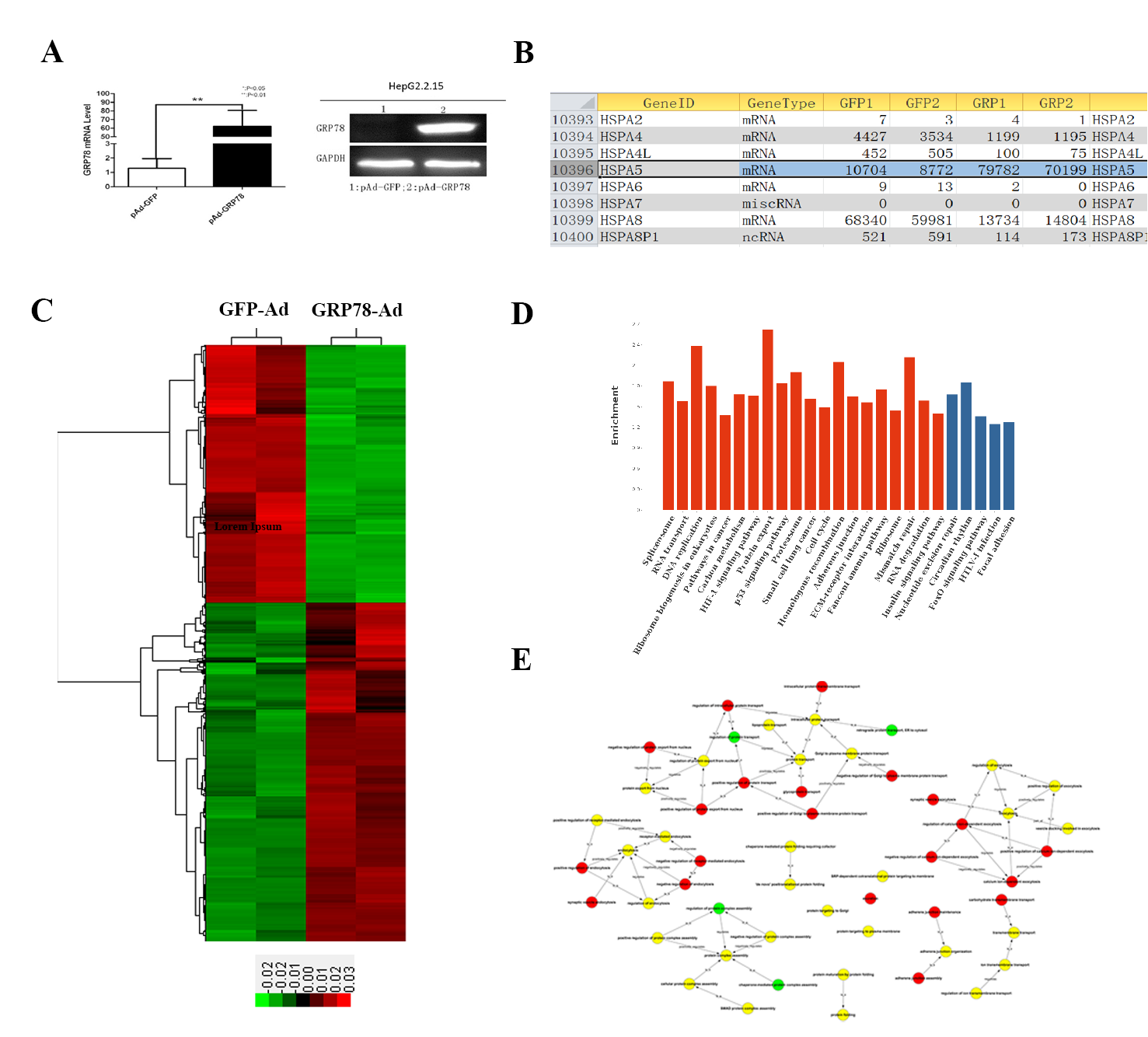


**Figure S6. Adenovirus overexpressing truncated or mutant GRP78.** A. Adenovirus overexpressing truncated GRP78 (Ad-GRP78-△ss). B. Adenovirus overexpressing the GRP78 ATPase domain (Ad-NBD) and substrate-binding domain (Ad-SBD). C. Adenovirus overexpressing the five mutant proteins of the GRP78 active site.

**
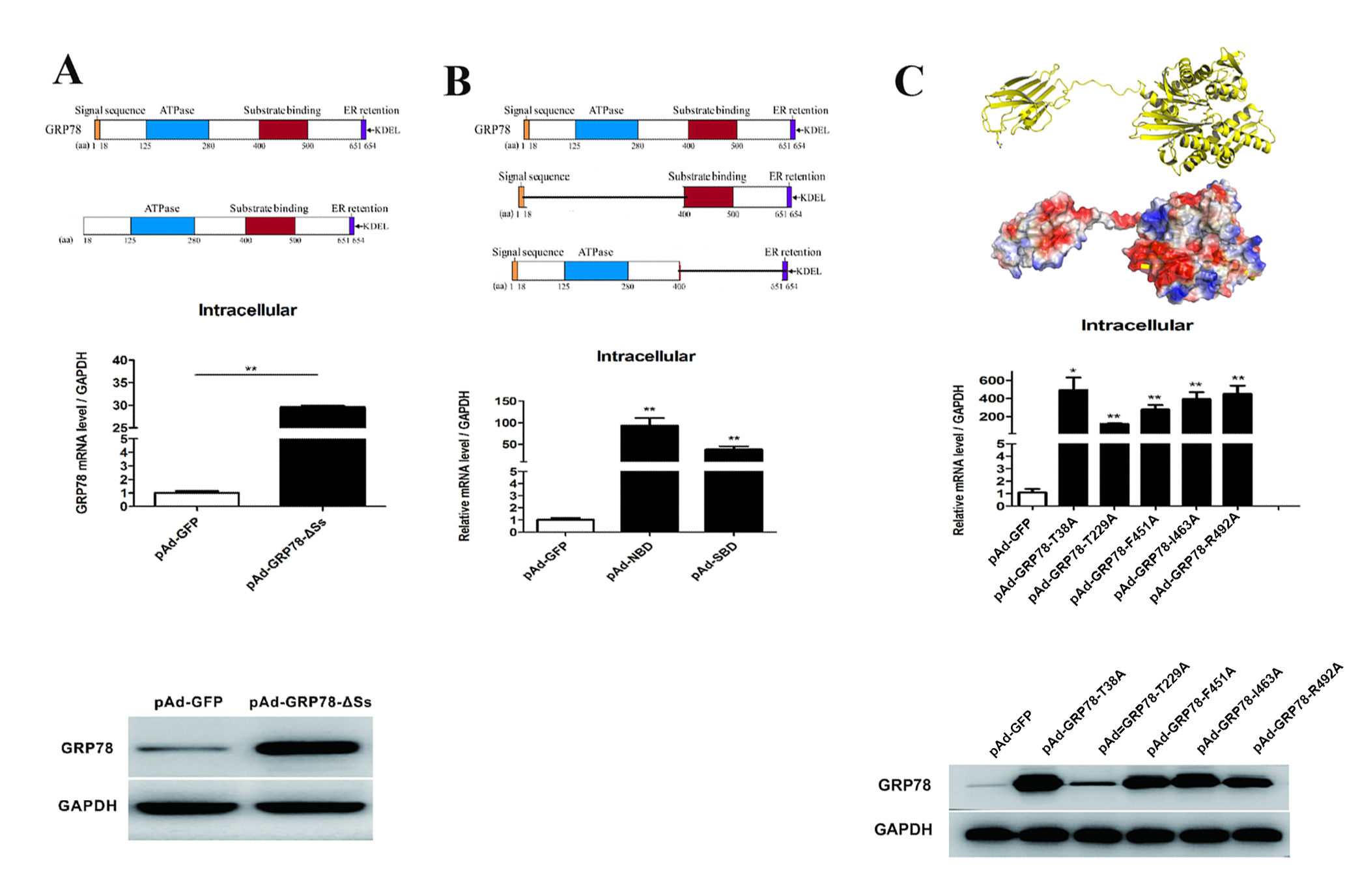
**

**Figure S7. The cytotoxicity of six peptides was detected by MTS cytotoxicity assay.** The results showed that these six peptides screened from phage display exhibited little effect on HepG2.2.15 cell viability.


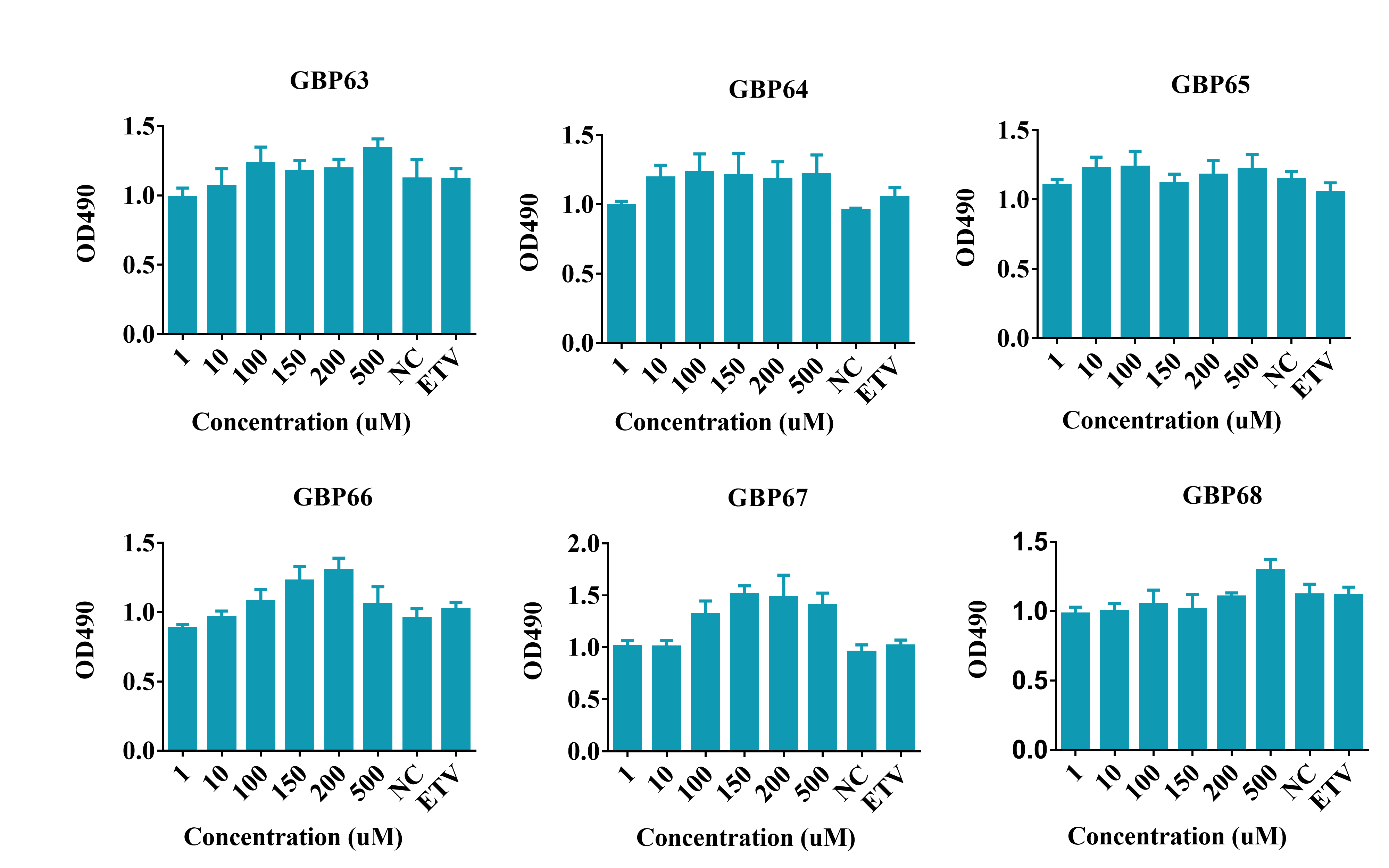


**Figure S8. HBV capsid and intact viral particles in HepAD38 supernatant and cytoplasm treated with glucose-regulated protein 68 GBP68 and entecavir (ETV).**


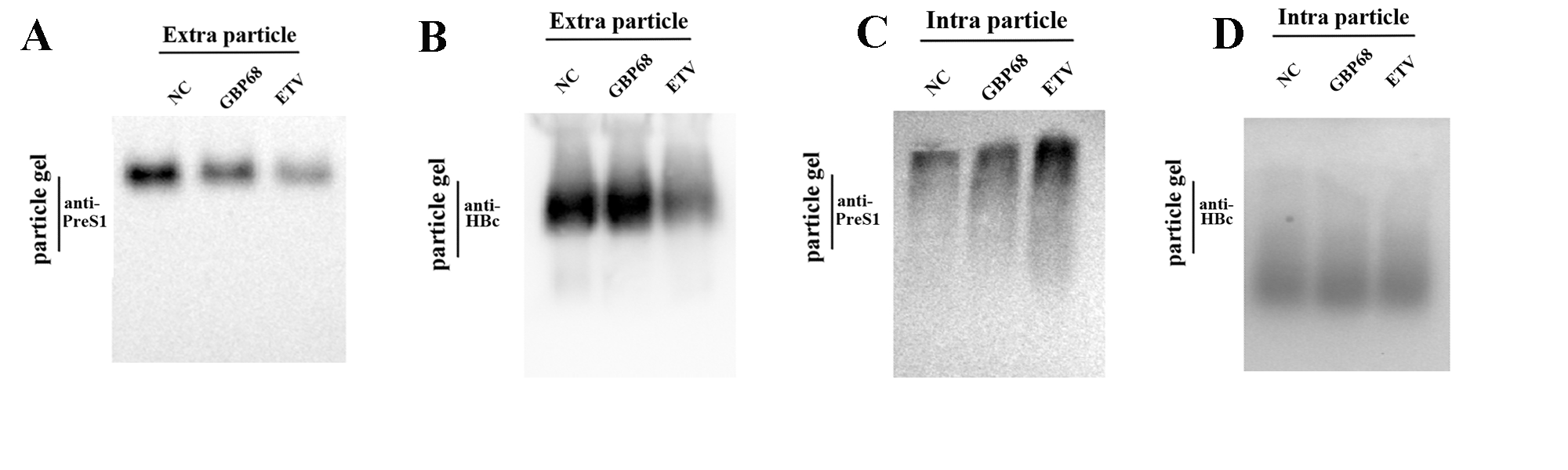


**Figure S9.** The complex structure model of the GRP78 and GBP63-68 complex by docking. The GRP78 structure model was cited from PDB (code: 6HAB), and the complex models were calculated using AutoDock Vina (http://vina.scripps.edu/). Cartoon and electrostatic potential surface representation of the overall structure of the complex. A saturated red color indicates Ø<−10 kiloteslas/e and a saturated blue indicates Ø>10 kiloteslas/e; T = 293 K. The residues involved in the H bond between GRP78 and the peptides are shown at the right of the figure. Among these complex, GRP78 interacted with GBP64 and GBP68 by 6 and 9 hydrogen bonds marked with Yellow dotted line in Figure S9, whereas, the other four peptides, including GBP63, GBP65, GBP66 and GBP67, there were no hydrogen bonds between GRP78 and theses peptides.


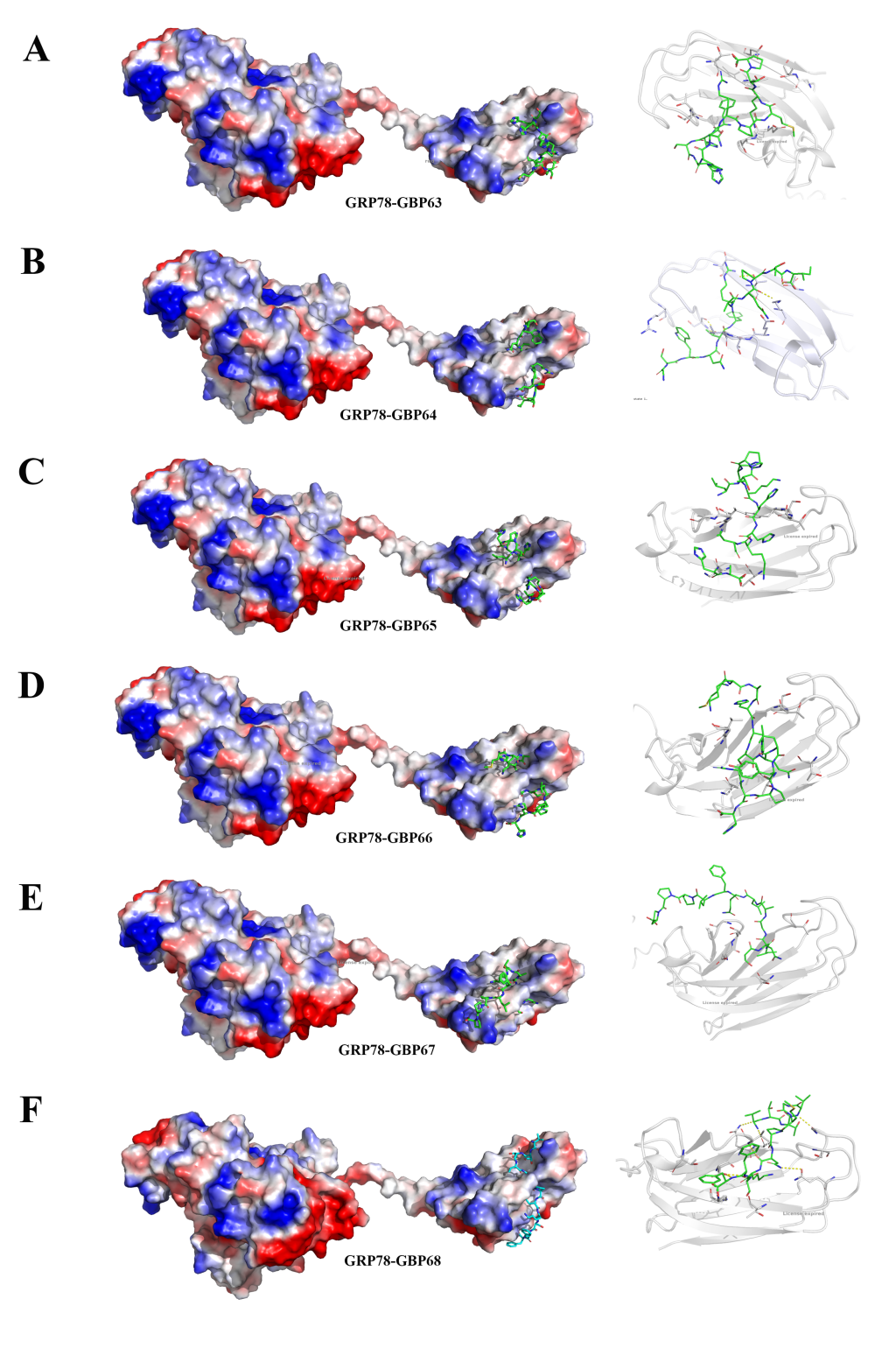


**Figure S10. The model of GRP78 regulates HBV particle secretion.** GRP78 promotes intact HBV particle secretion by directly interacting with preS1 located at the viral envelope. Some peptides could inhibit HBV particle excretion by binding to GRP78 with high affinity, which in turn reduced the inhibitory interaction between GRP78 and preS1/HBV particles.


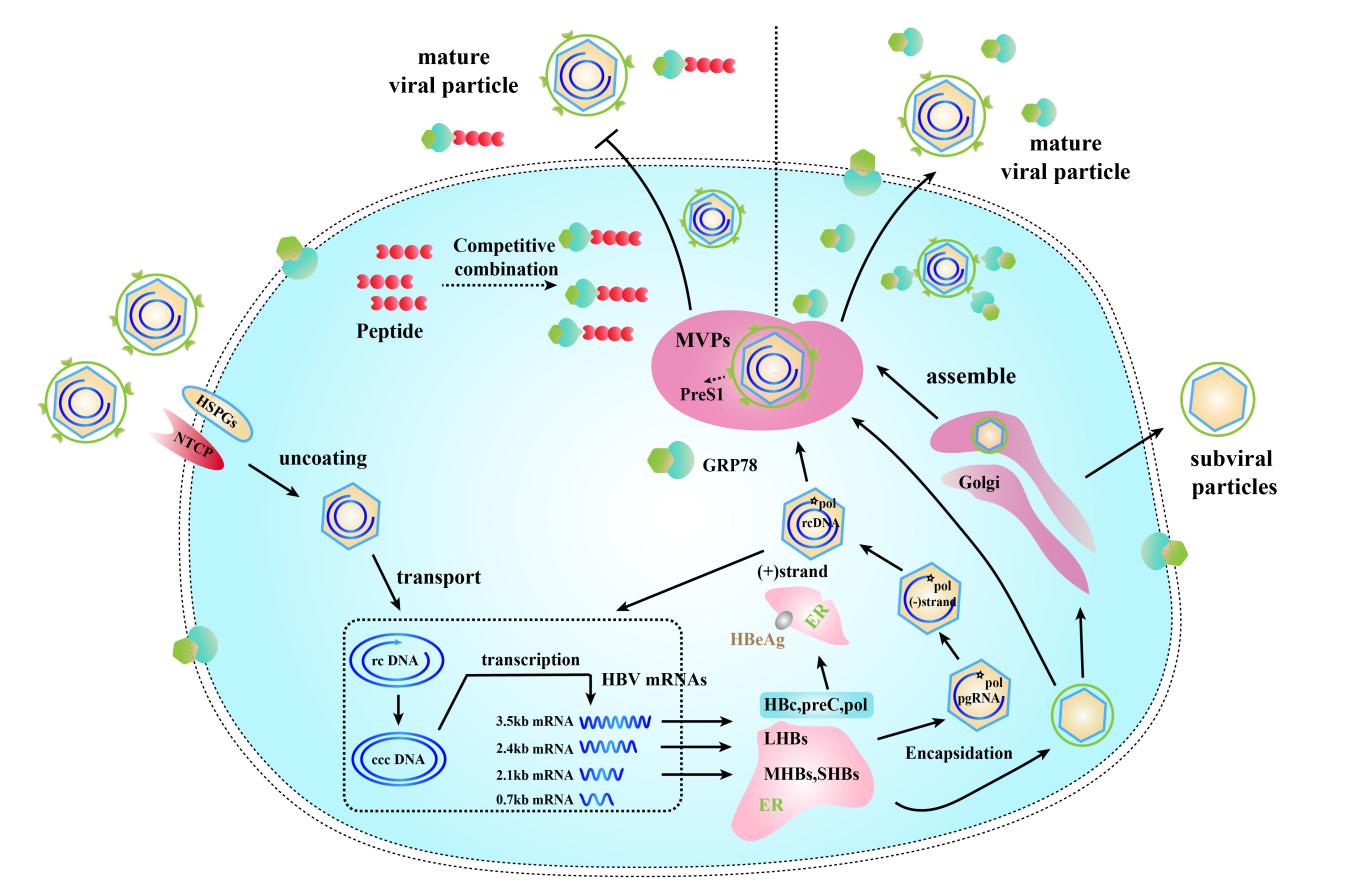

Supplement: Supplementary file 1 — Supporting information. [file JMV-95-0-s001.docx]
